# General Principles of Neuronal Co-transmission: Insights From Multiple Model Systems

**DOI:** 10.3389/fncir.2018.00117

**Published:** 2019-01-21

**Authors:** Erik Svensson, John Apergis-Schoute, Geoffrey Burnstock, Michael P. Nusbaum, David Parker, Helgi B. Schiöth

**Affiliations:** ^1^BMC, Department of Neuroscience, Functional Pharmacology, Uppsala University, Uppsala, Sweden; ^2^Department of Neurosciences, Psychology and Behaviour, University of Leicester, Leicester, United Kingdom; ^3^Department of Pharmacology and Therapeutics, University of Melbourne, Melbourne, VIC, Australia; ^4^Department of Neuroscience, Perelman School of Medicine, University of Pennsylvania, Philadelphia, PA, United States; ^5^Department of Physiology, Development and Neuroscience, Faculty of Biology, University of Cambridge, Cambridge, United Kingdom; ^6^Institute for Translational Medicine and Biotechnology, Sechenov First Moscow State Medical University, Moscow, Russia

**Keywords:** corelease, neurotransmitter complexity, neuromodulation, neuropeptides, colocalization

## Abstract

It is now accepted that neurons contain and release multiple transmitter substances. However, we still have only limited insight into the regulation and functional effects of this co-transmission. Given that there are 200 or more neurotransmitters, the chemical complexity of the nervous system is daunting. This is made more-so by the fact that their interacting effects can generate diverse non-linear and novel consequences. The relatively poor history of pharmacological approaches likely reflects the fact that manipulating a transmitter system will not necessarily mimic its roles within the normal chemical environment of the nervous system (e.g., when it acts in parallel with co-transmitters). In this article, co-transmission is discussed in a range of systems [from invertebrate and lower vertebrate models, up to the mammalian peripheral and central nervous system (CNS)] to highlight approaches used, degree of understanding, and open questions and future directions. Finally, we offer some outlines of what we consider to be the general principles of co-transmission, as well as what we think are the most pressing general aspects that need to be addressed to move forward in our understanding of co-transmission.

## Introduction

Co-localization reflects the presence of two or more substances within single synaptic terminals. This suggests that two or more transmitters can be released (co-release) to act as messengers (co-transmission). However, co-localization does not necessarily mean co-release or co-transmission: one or more co-localized substances may not be released, and if released they may lack functional effects, at least on the assayed neuron/circuit/behavior. Criteria to establish a co-transmitter match those for single transmitters, including evidence for their release, the existence of receptors, and inactivating and removal mechanisms under physiological conditions.

Co-localized substances have been defined in different ways, for example (neuro)transmitter or (neuro)modulator, slow/fast, ionotropic/metabotropic, or conventional/modulatory. The terminology was widely discussed in the past (Kupfermann, 1991), but there are exceptions to the various classification schemes. We know that single substances can serve different roles from transmitter, modulator, trophic factor, etc., depending on where and when they are released and the receptors to which they bind. Thus, amino acid transmitters can generate fast or slow, classical or modulatory, ionotropic or metabotropic, and signaling or trophic effects (Balazs, 2006). While amines and neuropeptides were considered to only generate slow, G protein receptor-mediated metabotropic responses, exceptions exist: the peptide FMRFamide activates ionotropic receptors (Cottrell, 1997), and 5-HT_3_ receptors are an ionotropic monoaminergic receptor (Barnes et al., 2009).

One general co-localization principle is that amino acid transmitters [glutamate, glycine, γ-aminobutyric acid (GABA), but also acetylcholine (ACh)] are contained in small synaptic vesicles (SSVs) located at active zones, monoamines are contained in small light or dense-core vesicles (SDCVs), and neuropeptides in large DCVs (LDCVs) located away from active zones (Hökfelt et al., 2003). In comparison to SSVs, DCV release is relatively poorly understood (Bulgari et al., 2018). While SNARE complexes and synaptic proteins are used for Ca^2+^-dependent DCV release, DCVs lack synapsins and do not form clusters at specialized release sites. This, together with their location away from the plasma membrane, tends to slow DCV release compared to SSV (Xia et al., 2009).

Co-transmission can be regulated by Ca^2+^-dependent signals generated by the frequency and duration of spiking, which can differentially release co-localized components (Peng and Horn, 1991; Verhage et al., 1991; Vilim et al., 1996a; de Wit et al., 2009). The typical effect is SSV release with low rates of presynaptic spiking, with recruitment of SDCV release as the duration and/or frequency of spiking increases. LDCV release tends to occur with higher frequency or burst spiking. High frequency spiking could in turn reduce SSV-mediated transmission as releasable vesicles become depleted, their release is actively depressed, or their postsynaptic effects undergo desensitization.

Transmitters can also co-localize in single vesicles (Jonas et al., 1998; Vilim et al., 2000; Merighi et al., 2011). While this suggests obligatory co-release, differential release can still result from kiss-and-run-like mechanisms (Xia et al., 2009). Even with full fusion, differential movement of molecules through fusion pores could temporally dissociate effects, and different diffusion rates to target receptors or enzymatic degradation that generates fragments with modified biological activity could also generate temporally and spatially specific signals (de Wit et al., 2009).

**Figure 1:**
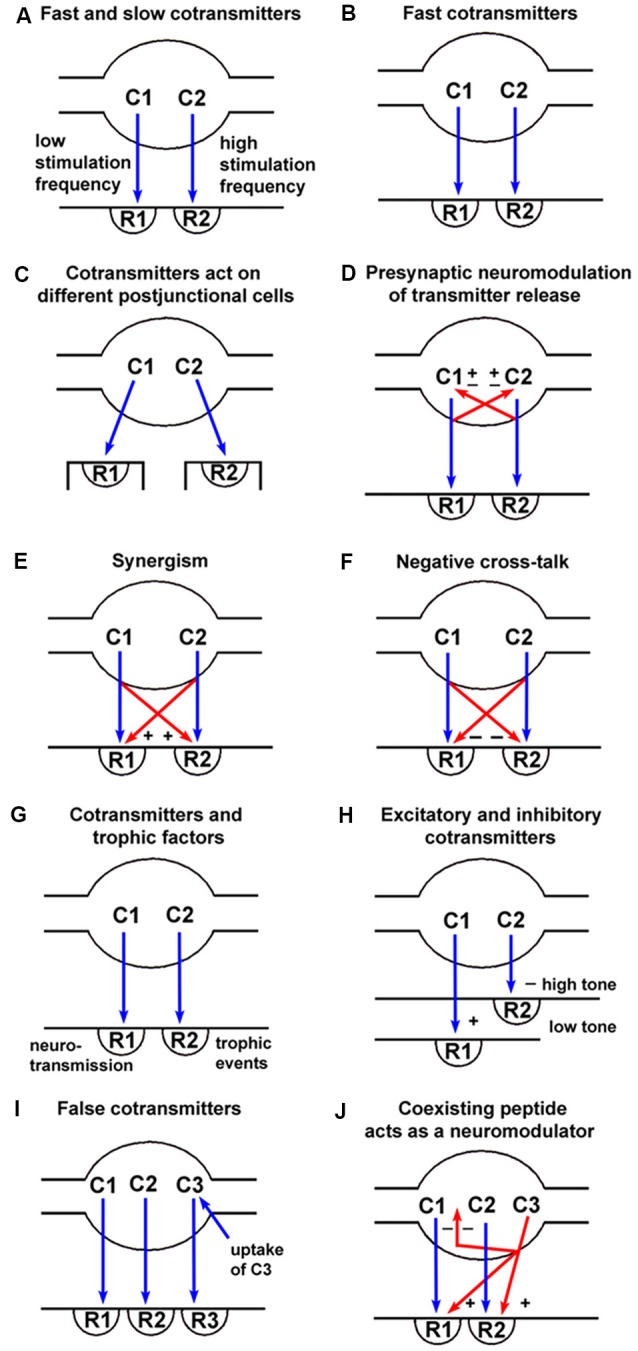
Spectrum of signaling variations offered by co-transmission (blue arrows = neurotransmission; red arrows = pre- or post-junctional neuromodulation). **(A)** Fast transmission is usually produced by small molecules (C1) released at low frequency nerve stimulation acting on ionotropic receptors (R1), whereas slow transmission is usually produced by release of peptides (C2) or other molecules at high frequency stimulation acting on G-protein-coupled receptors (R2). **(B)** Co-transmitters C1 and C2 can both be fast messengers acting *via* ionotropic receptors (R1 and R2). **(C)** Co-transmitters C1 and C2 act on receptors (R1 and R2) localized on different postjunctional cells. **(D)** Co-transmitters C1 and C2 not only act postjunctionally *via* R1 and R2 receptors but can also act as prejunctional modulators to either inhibit (−) or enhance (+) the release of C1 and/or C2. **(E)** Co-transmitters C1 and C2 act synergistically to enhance the combined responses produced *via* R1 and R2 receptors. **(F)** Co-transmitters C1 and C2 act to inhibit the responses evoked *via* R1 and/or R2 receptors. **(G)** Co-transmitter C1 evokes neurotransmission *via* R1 receptors, while C2 evokes long-term (trophic) responses of postjunctional cells *via* R2 receptors. **(H)** Co-transmitter C1 produces excitation *via* R1 receptors when the postjunctional smooth muscle target has low tone, with C2 having little influence; however, when the smooth muscle tone is high, the dominant response might be relaxation produced by C2 *via* R2 receptors. **(I)** Substance C3 is taken up by nerve terminals, rather than being synthesized and stored as is true for the co-transmitters C1 and C2. C3 can then be released on nerve stimulation to act on postjunctional R3 receptors. In these circumstances, C3 would be known as a “false transmitter.” **(J)** A coexisting substance C3 (often a peptide) can be synthesized and stored in a nerve, but not act directly *via* a postjunctional receptor to produce changes in postjunctional cell activity. It could, however, act as a prejunctional inhibitor (−) of the release of the co-transmitters C1 and C2, or as a postjunctional enhancer (+) of the responses mediated by R1 and R2. (Reproduced from Burnstock (2004), with permission from Elsevier).

A second general principle is that the co-localization and release of multiple transmitters provide flexibility to anatomically hard-wired circuits (see Figure 1 for examples of some of these effects). This is the reason usually given for the preponderance of transmitter substances (Marder, 2012). In addition, transmitter-receptor mismatches at classical synapses suggest longer-distance “volume transmission” as a mechanism working in parallel with conventional local or “wired” synaptic transmission to generate more diffuse effects (Fuxe et al., 2010). Various factors influence volume signaling. The extracellular space is a 3-dimensional matrix containing proteoglycans that determine tortuosity and diffusion distances (e.g., μm for monoamines to mm for neuropeptides). Diffusion direction and distance can also be influenced by receptor affinities, concentration gradients, uptake or breakdown mechanisms, charges on extracellular matrix molecules, or “tidal” effects caused, for example, by pressure differences resulting from cerebral blood flow (Krimer et al., 1998).

A third general principle is that a single transmitter can diverge to affect multiple receptors on multiple targets, while multiple transmitters can converge onto single effectors (Swensen and Marder, 2000; Brezina, 2010; Harris-Warrick and Johnson, 2010). These effects can change depending on the functional state of the targets. Co- or simultaneous release of transmitters will create a chemical “soup” around neurons that can alter individual transmitter effects (Brezina, 2010; Harris-Warrick and Johnson, 2010). Prior modulator release could also leave a background “modulatory tone” determined by the duration of 2nd messenger pathways and the phosphorylation state of targets that will influence subsequent effects. Rather than asking if modulatory systems interact, it seems more a question of how could they not. Analyzing one modulator at a time is of obvious utility in characterizing effects, but as with any experimental approach we need to ensure that we are not constraining system variables too tightly and as a result miss aspects essential to understanding normal function. While two or more ionotropic transmitters could interact through voltage and conductance changes, two (or more) modulators have multiple potential sites of interaction, including receptor binding, G protein activation, 2nd messenger cascades, and target effectors. When scaled up to the multiple transmitters and multiple targets in networks, the potential complexity is obvious. These interactions may be designed to constrain individual co-transmitter effects to prevent “over-modulation” (Harris-Warrick and Johnson, 2010; Marder et al., 2014), to decouple the divergent effects of a single modulator to produce net changes not possible with any single modulator (Brezina, 2010), or to modulate a shift from synaptic to a cellular driven activity (McClelland and Parker, 2017). However, we also have to consider that co-released transmitters do not necessarily interact (Yang et al., 1996; Blitz and Nusbaum, 1999).

## Historical Perspectives on Co-transmission

It was not until the early 1950’s that chemical transmission was fully accepted. John Eccles, who had been the most prominent critic of chemical transmission, used newly developed micropipettes and amplification equipment to examine his electrical hypothesis of transmission. Brock et al. (1952) recorded inhibitory postsynaptic potentials (IPSPs) in cat spinal cord motor neurons, an observation that negated his electrical hypothesis (Karl Popper had encouraged him to formulate his electrical hypothesis in a form that could be negated), and as a result he accepted chemical transmission (Parker, 2018). From almost 50 years of debate on the nature of central nervous system (CNS) transmission, the chemical transmission paradigm rapidly developed, principally through the work of Bernard Katz (1966) on the statistical nature of transmission and the role of Ca^2+^.

Much of the debate over chemical and electrical transmission was between Eccles and Henry Dale, one of the main proponents of chemical transmission during the first half of the 20th century. Eccles et al. (1954) coined the term “Dale’s Principle” when suggesting that motor neurons use the same transmitter at spinal cord collaterals to Renshaw cells as they do at the neuromuscular junction (Eccles et al., 1954): “In conformity with Dale’s principle that the same chemical transmitter is released from all the synaptic terminals of a neurone.” While not directly stated, this was subsequently taken to mean that neurons release a single transmitter at all of their synapses (the statement was probably not worded carefully because chemical transmission had only very recently been accepted, and with only two known transmitters the possibility of co-localization was probably not of obvious concern). Dale of course never stated this principle. Eccles et al referred to a lecture by Dale in 1935 in which he asked if identification of a peripheral chemical transmitter would “furnish a hint as to the nature of the transmission process at a central synapse? The possibility has at least some value as a stimulus to further experiment” (Dale, 1935). The subsequent erroneous interpretation of Dale’s Principle as one-neuron-one-transmitter led to claims by some that the principle had been invalidated when evidence of co-localization started to appear [this was even claimed long after co-localization was accepted (Nicoll and Malenka, 1998)]. As a result Eccles (1976) wrote “I proposed that Dale’s Principle be defined as stating that at all the axonal branches of a neurone, there was liberation of the same transmitter substance or substances”. Use of “substances” obviously removed any limit on how many transmitters were contained or released. This version of Dale’s principle has, however, been negated. Sossin et al. (1990) showed that the transmitter content differed in separate processes of single *Aplysia* neurons; Blitz and Nusbaum (1999) showed likely differential release of GABA and the peptide proctolin from separate terminals of a projection neuron in the crustacean stomatogastric ganglion (STG); Sulzer and Rayport (2000) showed that dopaminergic neurons co-release glutamate from only some terminals and Ludwig and Leng (2006) showed differential release from dendrites and synaptic terminals of single neurons.

Evidence for co-localization was actually found in vertebrates and invertebrates quite soon after chemical transmission was established (Abrahams et al., 1957; Burn and Rand, 1959; Gerschenfeld et al., 1960; Singh and Singh, 1966). For example, De Robertis and Pellegrino De (1961) showed the presence of two different types of vesicle in pineal gland terminals, Kerkut et al. (1967) showed the uptake of Dopa and 5-hydroxytryptamine (5-HT) in snail neurons and Su et al. (1971) showed co-release of adenosine 5′-triphosphate (ATP) and noradrenaline (NA) at sympathetic terminals. Jaim-Etcheverry and Zieher (1973) used the term “coexistence” for the location of NA and 5-HT in the pineal gland, Brownstein et al. (1974) showed anatomical evidence for co-localization in *Aplysia* neurons and Cottrell (1976) suggested that both ACh and serotonin were released from a single snail neuron to generate fast and slower responses, respectively. The development of immunohistochemistry directly demonstrated co-localization, leading to a plethora of studies showing multiple substances in single synaptic terminals (Hökfelt et al., 2000; Hökfelt, 2009), and co-localization of transmitters has now become the norm. For reviews detailing the changing concepts of co-localization and transmission see Burnstock (1976, 2014); Potter et al. (1981); Cuello (1982); Osborne (1983); Kupfermann (1991); Lundberg (1996) and Hökfelt (2009).

## Purinergic Co-transmission in the Autonomic and Central Nervous System

**Figure 2:**
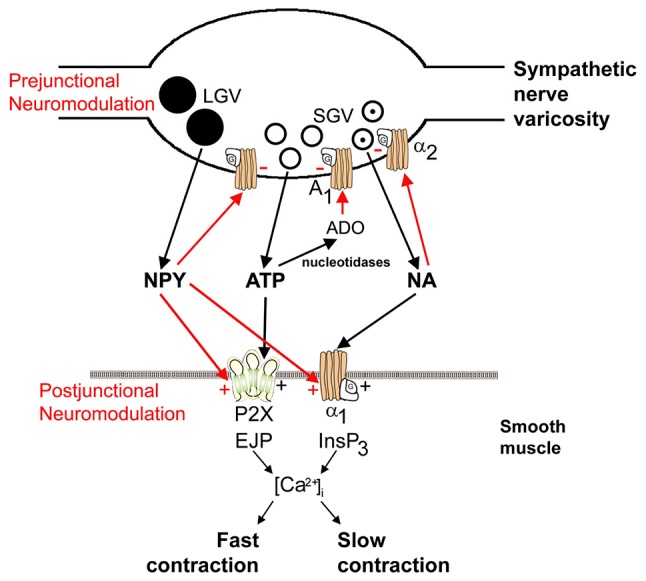
Schematic of sympathetic co-transmission. Adenosine 5’-triphosphate (ATP) and noradrenaline (NA) released from small granular vesicles (SGVs) act on P2X and α_1_ receptors on smooth muscle, respectively. ATP acting on inotropic P2X receptors evokes excitatory junction potentials (EJPs), an increase in intracellular calcium [(Ca^2+^)]_i_ and fast contraction; while metabotropic α_1_ adrenoceptors leads to production of inositol triphosphate (InsP_3_), an increase in (Ca^2+^)_i_ and slow contraction. Neuropeptide Y (NPY) stored in large granular vesicles (LGVs) acts after release both as a prejunctional inhibitory modulator of release of ATP and NA and as a postjunctional modulatory potentiator of the actions of ATP and NA. Soluble nucleotidases are released from nerve varicosities, and are also present as ectonucleotidases [reproduced from Burnstock and Verkhratsky (2010) with permission from Elsevier].

One of the first formal statements of the potential for co-transmission came from analyses of purinergic transmission (Burnstock, 1976), “Do some nerve cells release more than one transmitter?”. The purine nucleotide ATP had been identified as a signaling molecule in 1972 (Burnstock, 1972), and shown to be a co-transmitter with NA in sympathetic nerves see Figure 2 (Su et al., 1971; Nakanishi and Takeda, 1973; Burnstock, 1976, 1995; Langer and Pinto, 1976; Westfall et al., 1978). ATP has been subsequently found to co-localize with various transmitters in the peripheral and CNS (see Table 1 and reviews by Westfall et al., 1978; Burnstock, 2007; Wier et al., 2009; Hill-Eubanks et al., 2010; Hnasko and Edwards, 2012; Kennedy, 2015). For example, purinergic co-transmission is involved in the sympathetic control of arterial pressure in rats (Emonnot et al., 2006); as a co-transmitter with ACh in carotid body arterial chemoreceptors (Zapata, 2007); in the human carotid body where ACh, ATP and cytokines are co-released during hypoxia (Kåhlin et al., 2014); ATP and glutamate are released ectopically from vesicles along axons to mediate neurovascular coupling *via* glial calcium signaling (Thyssen et al., 2010); co-localized ATP and NA are involved in the sympathetic thermoregulatory response to cooling (Kozyreva et al., 2015); and ATP is released from dopaminergic neurons of the mouse retina and midbrain (Ho et al., 2015). For reviews describing the physiological significance of purinergic co-transmission see Burnstock (2004, 2014).

**Table 1:** Table showing transmitters co-localized with adenosine 5’-triphosphate (ATP) in the peripheral and central nervous systems (CNS).

**Peripheral nervous system**		
Sympathetic nerves	ATP + NA + NPY	Westfall et al. (1978) and Burnstock (1990)
Parasympathetic nerves	ATP + ACh +VIP	Hoyle (1994)
Sensory-motor	ATP + CGRP + SP	Burnstock (1993)
NANC enteric nerves	ATP +NO + VIP	Belai and Burnstock (1994) and Burnstock (2001)
Motor nerves (in early development)	ATP + ACh	Henning (1997)
**Central nervous system**		
Cortex, caudate nucleus	ATP + ACh	Richardson and Brown (1987)
Hypothalamus, locus coeruleus	ATP + NA	Sperlágh et al. (1997) and Poelchen et al. (2001)
Hypothalamus, dorsal horn, retina	ATP + GABA	Jo and Role (2002)
Mesolimbic system	ATP + DA	Krügel et al. (2013)
Hippocampus, dorsal horn	ATP + glutamate	Mori et al. (2001) and Fujii (2004)

*Modified from Abbracchio et al. (2009), with permission. ACh, acetylcholine; CGRP, calcitonin gene-related peptide; DA, dopamine; GABA, γ-aminobutyric acid; 5-HT, 5-hydroxytryptamine; NA, noradrenaline; NO, nitric oxide, NPY, neuropeptide Y; SP, substance P; VIP, vasoactive intestinal peptide*.

Changes in ATP co-transmission have also been implicated in pathological states. ATP is a co-transmitter with ACh in parasympathetic nerves supplying the diseased human bladder (Palea et al., 1993), and in sympathetic nerves in spontaneously hypertensive rats (Bulloch and McGrath, 1992). ATP also appears to be a co-transmitter involved in sympathetic pain, causalgia and sympathetic dystrophy, and is enhanced in inflammatory and stress conditions (Burnstock, 1996). Changes in transmitter co-localization in disease states or after injury is a common feature in different systems (see “Spinal Cord Modulation and Co-transmission” section below), which provides strong support for a specific functional role for co-transmission.

## The *Aplysia* Feeding Circuit

The impact of co-transmission on circuit activity has been analyzed in detail in invertebrate systems. These systems offer use conventional electrophysiological techniques to identify and determine the function of co-transmitters in physiologically identified neurons in defined neuronal circuits (Kupfermann, 1991; Nusbaum et al., 2001, 2017; Cropper et al., 2018).

The *Aplysia* feeding circuit has provided important insights into the functional role of co-transmission (Brezina, 2010). Brezina et al. (1996) and have provided a detailed analysis of how the peptide co-transmitters small cardioactive peptide and myomodulin modulate muscle contractions evoked by their small molecule co-transmitter ACh released from motor neurons onto muscle controlling feeding behavior. More significantly, this analysis has shown that transmitter-specific divergent and convergent interactive effects of the modulators on targets involved in excitation-contraction coupling (e.g., calcium channels, potassium channels, relaxation rate) can evoke novel effects not seen with either modulator individually.

**Figure 3:**
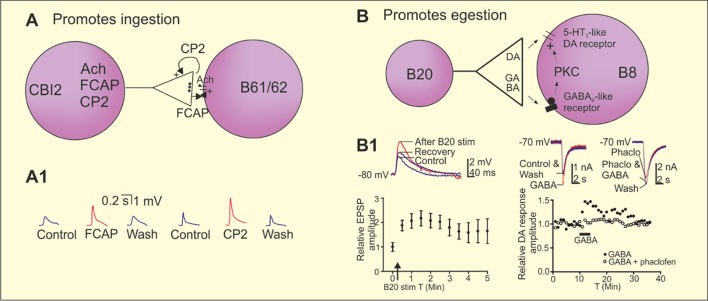
Co-transmission in the regulation of ingestion and egestion in the *Aplysia* feeding circuit. **(A)** Repeated stimulation of the cerebral buccal interneuron 2 (CBI-2) progressively induces the ingestive motor programs in *Aplysia*. The command neuron CBI-2 uses acetylcholine (Ach) as its fast-excitatory neurotransmitter onto the motor neurons B61/62. CBI-2 also co-localize the two neuropeptides feeding circuitry activating peptide (FCAP) and cerebral peptide 2 (CP-2). Both peptides are released by high frequency stimulation of CBI-2 and contributes to post-tetanic potentiation (PTP) of the fast-cholinergic transmission at the CBI-2 to B61/62 synapses, however by different mechanisms. CP-2 is acting presynaptically and FCAP postsynaptically. **(A1)** Recording traces showing the potentiation of the cholinergic EPSP by FCAP and CP-2 in B8. **(B)** Repeated stimulation of the esophageal nerve and high frequency stimulation of the interneuron B20 that co-localizes dopamine and γ-aminobutyric acid (GABA) induces egestion and a short-term potentiation of the fast dopaminergic EPSPs between B20 and the motor neuron B8. Dopamine acts on a 5-HT_3_-like receptor and GABA contributes to the potentiation of the B20 to B8 EPSP by a postsynaptic mechanism that involves activation a GABA_B_ receptor of protein kinase C (PKC). **(B1)** High frequency stimulation of B20 potentiates the dopaminergic EPSPs in B8. GABA potentiated fast dopaminergic responses in B8 and the effect is blocked by the GABA_B_ receptor antagonist phaclofen. Adapted from Koh et al. (2003), Koh and Weiss (2005) and Svensson et al. (2014).

*Aplysia* can generate two antagonistic feeding behaviors, namely ingestion and egestion. Repetitively stimulating the command neuron cerebral-buccal interneuron 2 (CBI-2) can activate the feeding central pattern generator (CPG) in the buccal ganglion to progressively produce ingestive motor programs “repetition priming of motor activity” (Cropper et al., 2014). CBI-2 co-localizes ACh, feeding circuit activating peptide (FCAP), and cerebral peptide 2 (CP-2). The neuropeptide actions diverge (CP-2 appears to act presynaptically while FCAP acts postsynaptically at the same cholinergic synapses) but their combined actions converge to potentiate fast cholinergic EPSPs in motor neurons B61/62 (Figure 3A; Koh et al., 2003; Koh and Weiss, 2005). Co-transmission thus allows distinct signals to be sent that regulate “conventional” transmission.

Egestion is activated by repetitive stimulation of the esophageal nerve, which induces a short-term potentiation of synaptic transmission between interneuron B20 and the follower motor neuron B8. B20 co-localizes GABA and dopamine (Díaz-Ríos and Miller, 2005; Svensson et al., 2014). Dopamine acts as a fast-excitatory transmitter by acting on a 5-HT_3_-like receptor. GABA does not have any fast direct effect at this synapse, but can potentiate dopaminergic responses by acting on a GABA_B_ receptor and subsequently activating protein kinase C (PKC; Figures 3B,B1; Svensson et al., 2014). This is an example where the “conventional” transmitter (GABA) evokes a modulatory effect and the “modulatory” transmitter (dopamine) a conventional effect. This effect of GABA is considered to be an example of intrinsic modulation by a co-transmitter as it modulates the circuit to which it belongs. Co-localization of dopamine and GABA is common. It also occurs in lamprey, and in dopaminergic neurons of the mammalian substantia nigra pars compacta and ventral tegmental area (VTA; Barreiro-Iglesias et al., 2009; Tritsch et al., 2012; Berrios et al., 2016; Ntamati and Lüscher, 2016), and in hypothalamic feeding circuits (see below).

## Co-transmission Consequences in the Decapod Crustacean Stomatogastric Nervous System

### Early Contributions

**Figure 4:**
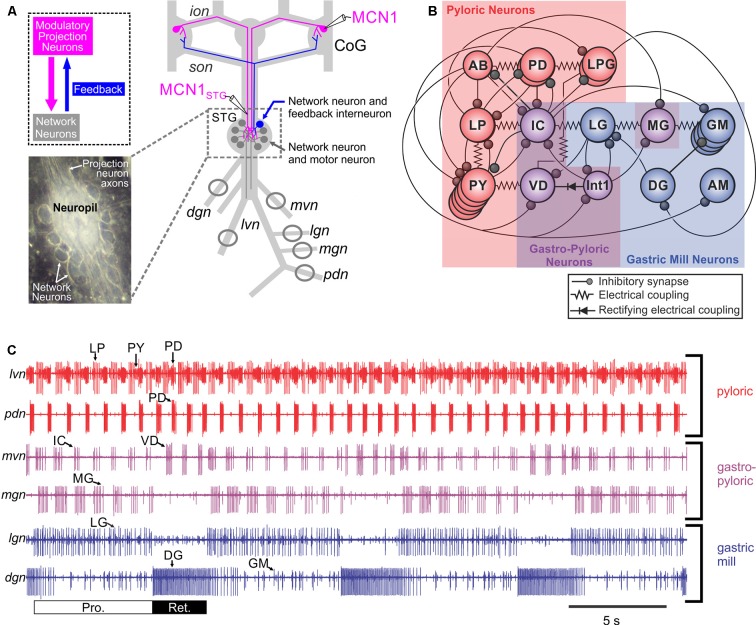
The crab *Cancer borealis* stomatogastric nervous system (STNS). **(A)** Schematic of the isolated STNS of the crab *Cancer borealis*. The inset shows a whole-mount image of the desheathed stomatogastric ganglion (STG) under dark-field illumination (anterior, top; posterior, bottom). As it is evident in both the schematic and the inset, the 26 neuronal somata form a single layer surrounding the neuropil. Circles on nerves indicate recording sites for traces shown in part **(C)**. **(B)** Schematic of the gastric mill and pyloric circuit. The arrangement of neurons in the schematic represents the relative timing of activity for each neuron during the gastric mill and pyloric rhythms. Specifically, the neurons that exhibit pyloric rhythm-timed activity (“pyloric neurons” and “gastropyloric neurons”) are displayed such that the top-row neurons are co-active, followed by the middle-row neurons and then the bottom-row neurons, after which the top-row neurons are again active. The neurons that exhibit gastric mill rhythm (GMR)-timed activity (“gastric mill neurons” and “gastropyloric neurons”) are displayed such that the top-row neurons are co-active and burst in alternation with the bottom-row neurons. As shown, there are eight gastric mill circuit neuron types, one of which is present as four apparently equivalent copies (GM neurons). All eight neuron types contribute to gastric mill pattern generation, whereas only two (LG and Int1) are also rhythm generator neurons (Coleman et al., 1995; Bartos et al., 1999; Saideman et al., 2007). There are seven pyloric circuit neuron types, including the rhythm generator (“pacemaker”) group AB/PD/LPG (Marder and Bucher, 2007). Three of these neuron types are present as multiple, apparently equivalent copies (PD: 2; LPG: 2; PY: 5). **(C)** Simultaneous extracellular nerve recordings of the gastric mill and pyloric rhythms during tonic stimulation of the modulatory projection neuron modulatory commissural neuron 1 (MCN1) are shown. The pyloric rhythm exhibits a rhythmically repeating triphasic pattern (for example, lateral ventricular nerve (*lvn*): PD, LP, PY) that is continuously active, *in vivo* and *in vitro*, with a cycle period of ~1 s. The GMR (cycle period ~10–20 s) is silent except when driven by modulatory neurons (for example, MCN1), which themselves require activation *in vivo* and *in vitro*. It is a rhythmically repeating biphasic pattern, consisting of teeth protraction (Pro.) and teeth retraction (Ret.), which drives the motor response (chewing). Note that some neurons exhibit activity patterns time-locked to both rhythms (gastropyloric neurons). CoG, commissural ganglion; *dgn*, dorsal gastric nerve; *ion*, inferior oesophageal nerve; *lgn*, lateral gastric nerve; *mgn*, medial gastric nerve; *mvn*, medial ventricular nerve; *pdn*, pyloric dilator nerve; *son*, superior oesophageal nerve; *stn*, stomatogastric nerve.Panels **(A)** and **(C)** are adapted with permission Nusbaum et al. (2017), Nature Reviews Neuroscience. Panel **(B)** is adapted with permission from Nusbaum and Beenhakker (2002), Nature. STG photo courtesy of Marie Suver, New York University, USA, and Wolfgang Stein, Illinois State University, USA.

Significant insight into the cellular and circuit effects of co-localized transmitters has been obtained in the isolated decapod crustacean stomatogastric nervous system (STNS: Figure 4; Nusbaum et al., 2001, 2017). The STNS, an extension of the decapod CNS, is composed of four ganglia plus their connecting and peripheral nerves (Figure 4; Nusbaum et al., 2001; Marder and Bucher, 2007). The ganglia include the paired commissural ganglia (CoGs: each containing ≥500 neurons) and the unpaired oesophageal (OG: 15–20 neurons) and stomatogastric (STG: 25–30 neurons) ganglia; the number of neurons per ganglion is species-specific. These ganglia contain several CPG circuits which regulate the ingestion and processing of food by the striated muscles of the foregut. As is common for CPGs, these circuits continue to operate in the isolated STNS, maintained in physiological saline, in a manner similar to their activity *in vivo* (Heinzel et al., 1993; Diehl et al., 2013; Yarger and Stein, 2015). The gastric mill (chewing) and pyloric (pumping and filtering of chewed food) circuits, both located in the STG, are extensively characterized (Figure 4; Marder and Bucher, 2007; Stein et al., 2007; Marder, 2012; Nusbaum et al., 2017). Despite each of these circuits being composed of a small number of neurons, they are remarkably flexible in their response to different modulatory influences and can generate many different versions of the gastric mill and pyloric rhythms.

Co-transmission studies in the STNS, primarily in the crab *Cancer borealis*, have involved manipulating the activity of identified modulatory projection neurons and sensory neurons which influence the gastric mill- and pyloric circuits (see Table 2: Katz and Harris-Warrick, 1990; Blitz and Nusbaum, 1999; Blitz et al., 1999; Wood et al., 2000; Wood and Nusbaum, 2002; Christie et al., 2004; Stein et al., 2007; DeLong et al., 2009a,b). As summarized below, these studies, and related ones in the lobster STNS (Meyrand et al., 2000; Thirumalai and Marder, 2002; Kwiatkowski et al., 2013), revealed that co-transmitting neurons provide many degrees of freedom to circuit outputs.

**Table 2:** Identified co-transmitter neurons that influence stomatogastric ganglion (STG) circuits.

Identified neuron	Co-transmitters	Target circuit(s)	Key action
^1^GPR	ACh, 5HT, AST-A	PR, GMR	PR/GMR: M/A
^1^MPN	GABA, Proct	PR, GMR	PR: M/A; GMR: I
^2^GN1/2	GABA, CCK-LI, FXRFamide-LI	PR, GMR	PR/GMR: M/A
^1^MCN1	GABA, Proct, CabTRP Ia	PR, GMR	PR: M/A; GMR: A
^1,3^IVN	HA, FXRFamide-LI	PR, GMR, OR	PR: I; GMR: A; OR: M
^4^PS	HA, Crust-MS*	PR, GMR, OR	PR/GMR/OR: M/A

Legend: M, modulates; A, activates; I, inhibits; LI, like-immunoreactivity; *Crust-MS is a FXRF-like peptide. ^1^C. borealis; ^2^H. gammarus; ^3^P. interruputs; ^4^H. americanus. PR, pyloric rhythm; GMR, gastric mill rhythm; OR, oesophageal rhythm.

### Applied vs. Endogenously Released Neuropeptides

**Figure 5:**
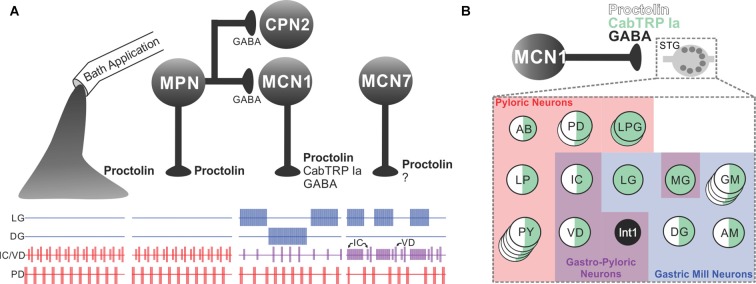
The microcircuit response to peptidergic neuron activity is not necessarily mimicked by bath application of that neuropeptide. **(A)** Extracellular recordings of identified neurons in the crab *Cancer borealis* STG, which are active during the GMR (LG and DG neurons), pyloric rhythm (PD neuron) or both rhythms (IC and VD neurons). In the isolated crab STG, bath-applied proctolin (far left set of responses) selectively excites the pyloric rhythm (Marder et al., 1986; Nusbaum and Marder, 1989a). This action mimics the response to activation of only one [modulatory proctolin neuron (MPN)] of the three proctolinergic projection neurons that innervate the STG (MPN, MCN1 and MCN7), even though MPN also contains a small-molecule co-transmitter (GABA; Blitz et al., 1999). As indicated, MPN also inhibits two projection neurons [MCN1 and commissural projection neuron 2 (CPN2)] by releasing GABA from a separate axon projecting to a separate location (CoG; Blitz and Nusbaum, 1997, 1999). The other two proctolinergic projection neurons (MCN1 and MCN7) also influence STG microcircuit activity but elicit activity patterns from the circuit neurons that are distinct from proctolin bath application (Coleman and Nusbaum, 1994; Blitz et al., 1999). MCN1-released *C. borealis* tachykinin-related peptide Ia (CabTRP Ia) and GABA are pivotal for MCN1 activation of the GMR, whereas its release of CabTRP Ia and proctolin dominates its excitation of the pyloric rhythm (see part **B**). The MCN7 actions on these rhythms result partly from proctolin and probably also from one or more yet-to-be-identified co-transmitters (indicated by “?”). In the figure, pyloric rhythm activity is shown in red; GMR activity is shown in blue; gastropyloric activity is shown in purple. **(B)** In the crab STG, MCN1 influences all pyloric, gastropyloric and gastric mill neurons. The figure shows a representation of responsiveness of each STG circuit neuron to the MCN1-released co-transmitters proctolin (white w/black border), CabTRP Ia (green) and GABA (black; Swensen and Marder, 2001; Stein et al., 2007). Examples of convergent peptide co-transmitter action (proctolin and CabTRP Ia), selective peptide co-transmitter action (CabTRP Ia) and selective GABA action are shown. In some cases, the STG neuron only responds to the indicated co-transmitter (or co-transmitters; for example, Int1). In other cases, the STG neuron does respond to an additional co-transmitter but not when it is released from MCN1 (for example, LG responds to applied GABA but not GABA released from MCN1). No information is available regarding whether these co-transmitters are colocalized to all MCN1 terminals or are localized to separate terminals for their release. Panel **(A)** is adapted with permission from Nusbaum et al. (2001), Elsevier. Panel **(B)** is adapted with permission from Nusbaum et al. (2017), Nature Reviews Neuroscience.

As discussed in several sections of this review, neuropeptides, the largest and most diverse class of neurotransmitters, are commonly present as co-transmitters (Merighi et al., 2011; Taghert and Nitabach, 2012; van den Pol, 2012; Nusbaum et al., 2017; Nässel, 2018). Due to the challenges associated with studying peptidergic (co)transmission, neuropeptide actions have often been studied *via* their exogenous application to the nervous system. This approach has been considered a reasonable proxy for peptidergic transmission because neuropeptide release is envisioned to act *via* volume transmission (see above). There are, however, several reasons why exogenously applied and neurally-released peptides would not necessarily have equivalent actions (Nusbaum et al., 2017), one of which is the interaction of their effects with those of co-released transmitters. The relative influence of exogenously applied and endogenously released peptides was directly determined in *C. borealis* by comparing the pyloric rhythm response to bath-applied proctolin (RYLPT) with that evoked by separate stimulation of the three proctolinergic projection neurons [modulatory proctolin neuron (MPN), modulatory commissural neuron 1 (MCN1), MCN7] that innervate the STG (Figure 5). Proctolin application and stimulating each proctolin neuron all excite/modulate the pyloric rhythm, but only the pyloric rhythm configured by MPN activity was comparable to proctolin application (Nusbaum and Marder, 1989a,b; Blitz et al., 1999). MCN1 or MCN7 stimulation elicited pyloric rhythms that were different from that driven by MPN/proctolin and from each other (Blitz et al., 1999; Wood et al., 2000). Interestingly, the match between MPN stimulation and proctolin application occurred despite the fact that MPN co-releases GABA (Blitz and Nusbaum, 1999; see “Convergent and Divergent Co-transmission” section below).

### Shared Co-transmitters and Circuit Targets

Neurons which use the same co-transmitters and contact the same circuit can nevertheless elicit distinct responses from that circuit. This was established in the crab STG by comparing the influence of MPN and MCN1, two projection neurons which both use proctolin and GABA as co-transmitters (Figure 5; Blitz et al., 1999). MCN1 also contains a second peptide co-transmitter, CabTRP Ia (Christie et al., 1997; Blitz et al., 1999). When CabTRP Ia actions were suppressed, MPN and MCN1 still elicited distinct pyloric motor patterns (Wood et al., 2000). Motivated by earlier studies in *Aplysia* that documented how effectively extracellular peptidase activity can regulate peptidergic actions (Sigvardt et al., 1986; Owens et al., 1992; Rothman et al., 1992), a proctolin peptidase inhibitor was applied to the STG, resulting in convergence of the MPN- and MCN1-elicited pyloric rhythms (Coleman et al., 1995; Wood and Nusbaum, 2002). These results suggested that the peptidergic action of these two neurons on the pyloric circuit was being differently sculpted, at least partly, by MPN and MCN1 releasing (a) comparable amounts of proctolin at different distances from its sites of extracellular cleavage by peptidase activity, and/or (b) different amounts of proctolin per action potential (Wood and Nusbaum, 2002).

### Convergent and Divergent Co-transmission

The concepts of convergent and divergent signaling were established in the earliest co-transmission studies (Jan et al., 1979, 1980; Hökfelt et al., 1980; Lundberg et al., 1981; Jan and Jan, 1982). These seminal studies also established that neurally-released peptides can diffuse to activate receptors well beyond the boundaries of the synaptic cleft (Jan and Jan, 1982). Soon thereafter, convergent co-transmission was revealed in the arthropod (insect and crustacean) neuromuscular system (Adams and O’Shea, 1983; Bishop et al., 1987), while divergent co-transmission was documented in several systems, ranging from the *Aplysia* neuroendocrine and neuromuscular systems to the rodent thalamus (Mayeri et al., 1985; Sigvardt et al., 1986; Vilim et al., 1996a,b; Vilim et al., 2000; Koh et al., 2003; Sun et al., 2003).

There are many potential consequences of convergent and divergent co-transmission for circuit operation. For example, each could have linear or non-linear actions on circuit neurons and/or the circuit output. Additionally, different neurons in the same circuit could be targets of convergence or divergence (Wood et al., 2000; Thirumalai and Marder, 2002; Stein et al., 2007; Nusbaum et al., 2017). Given the myriad potential degrees of freedom provided by co-transmission, elucidating the cellular and synaptic mechanisms underlying their impact on circuit activity is facilitated by intracellular access to all circuit neurons.

One of the earliest studies of convergent peptidergic co-transmission examined the MCN1 (proctolin, CabTRP Ia) influence on the crab pyloric rhythm (Wood et al., 2000). This study compared the pyloric rhythm in response to normal MCN1 input relative to its input after CabTRP Ia actions were suppressed, and showed that both peptides contributed to the effects of MCN1 and had comparable actions on each pyloric circuit neuron. Their relative influence, however, appeared to be unequal because when CabTRP Ia actions were suppressed the pyloric cycle frequency and activity level of pyloric neurons remained >50% larger than without MCN1 stimulation. This interpretation, however, was inconclusive because proctolin and CabTRP Ia activate the same voltage-dependent ionic current (called I_MI_) in STG neurons, albeit by binding to different receptors (Swensen and Marder, 2000, 2001; DeLong et al., 2009b). This convergence suggested a ceiling effect due to the combined action of both peptides activating all available I_MI_ so that, when CabTRP Ia actions were suppressed, more I_MI_ might have been available for proctolin to activate. I_MI_ activation can be limiting in STG neurons (Garcia et al., 2015), but in this case there appeared to be no such limitation because with the CabTRP Ia actions present, the peptidergic excitation of several pyloric neurons by MCN1 was increased in the presence of a proctolin peptidase inhibitor relative to normal MCN1 stimulation (Wood and Nusbaum, 2002).

Divergent co-transmission can act on separate circuit neurons and separate circuits, providing several different types of functional flexibility. These include: (1) influencing separate target neurons in the same circuit to collectively affect the circuit-level response; (2) selectively influencing release of a subset of co-transmitters; (3) enabling temporally separate responses from different circuit neurons; and (4) displaying spatially separate co-transmitter actions that influence different circuits. In the American lobster (*Homarus americanus*) STNS, a projection neuron that innervates the STG contains the peptides RPCH and CabTRP Ia (Thirumalai and Marder, 2002). This is the only source of CabTRP Ia in the *H. americanus* STG neuropil, while there is one additional RPCH neuron innervating this STG. Co-applying these peptides, but not their separate application, to the isolated STG activates the complete pyloric rhythm sequence of AB/PD, LP and PY neuron bursting. Applying them separately reveals that their actions converge to excite the pyloric pacemaker group AB/PD, but RPCH only activates the LP neuron while CabTRP Ia activates only the PY neurons. Divergent actions also result from the influence of the muscle stretch-sensitive sensory neuron GPR, which contains 5-HT, ACh and AST-A peptide (A-type allatostatin; Beltz et al., 1984; Katz et al., 1989; Skiebe and Schneider, 1994; Szabo et al., 2011). GPR primarily uses divergent co-transmission to modulate the pyloric- and GMRs, with the caveat that its AST actions remain to be determined. GPR has convergent 5-HT and ACh actions on only one pyloric circuit neuron (IC neuron), while it evokes only cholinergic EPSPs in the VD neuron and only serotonergic modulatory responses in the remaining circuit neurons (AB, PD, LPG, LP, PY). Collectively, these actions modify the pyloric cycle frequency and the pattern of the ongoing rhythm (Katz and Harris-Warrick, 1989, 1990). The relative influence of the cholinergic EPSPs and 5-HT modulation remains to be determined, but the latter effect clearly outlasts the former. As a further wrinkle, GPR that is rhythmically active *in situ* with a cycle period (~10–20 s), that is much slower than the pyloric rhythm (~1 s), due to the GMR-timed stretch of the muscles that GPR innervates. Thus, its pyloric circuit modulation waxes and wanes along with the gastric mill cycle motor pattern.

### Selective Regulation of Co-transmitter Release

GPR also influences an ongoing MCN1-driven GMR, prolonging the retractor phase without altering the protractor phase duration (Beenhakker et al., 2005). This GPR action again results from divergent co-transmission, as it is suppressed by a 5-HT receptor antagonist (DeLong et al., 2009a). GPR has divergent co-transmitter actions on all three GMR generator neurons [LG, Int1, MCN1_STG_ (axon terminals in STG)] but only its 5-HT-mediated inhibition of MCN1_STG_ is effective during this GMR (Beenhakker et al., 2005; DeLong et al., 2009a). Interestingly, this 5-HT inhibition of MCN1_STG_ selectively suppresses the MCN1 peptidergic (CabTRP Ia) excitation of LG without altering its GABAergic action onto Int1. The ability to separately change the amount of each co-transmitter released from a neuron can increase the functional flexibility of a co-transmitting neuron in a state-dependent manner.

Temporally distinct effects underlie the MCN1 projection neuron activation of the GMR, a result of its divergent co-transmitter excitation of the GMR generator neurons LG and Int1 (Coleman et al., 1995; Stein et al., 2007). During the GMR, MCN1 releases its co-transmitters during the retraction phase, but while Int1 is active during retraction, LG is active during protraction. This sequential activation is accomplished in part by MCN1 eliciting a fast, ionotropic (GABA) excitation of Int1 and a slow, metabotropic (CabTRP Ia) excitation of LG (see also Schöne et al., 2014).

Spatially separate co-transmitter actions on different circuits have also been demonstrated. In addition to its proctolinergic excitation of the pyloric rhythm in the crab STG (Nusbaum and Marder, 1989a,b), MPN projects an axon to each CoG where it produces a GABAergic inhibition of the projection neurons MCN1 and CPN2 (Blitz and Nusbaum, 1997, 1999). Although MCN1/CPN2 are excited by applied proctolin, they do not respond to MPN stimulation when the GABAergic inhibition is pharmacologically suppressed, suggesting that MPN does not release proctolin in the CoG (Blitz and Nusbaum, 1999; Marder, 1999). The MPN actions in the CoG ensure a proctolin-specific modulation of the pyloric rhythm in the STG, and prevent MCN1/CPN2 activation of the GMR. Similarly, the IVN (*C. borealis*, *Panulirus interruptus*)/PS (*H. americanus*) neuron has divergent co-transmitter actions in the STG and CoG which directly and indirectly influence the STG circuits, as well as influencing the oesophageal circuit in the CoG (Russell and Hartline, 1981; Sigvardt and Mulloney, 1982; Claiborne and Selverston, 1984; Marder and Eisen, 1984; Christie et al., 2004; Kwiatkowski et al., 2013).

### Species-Specific Co-transmission

While there is considerable conservation of structure and function in the STNS across species of lobster, crab, crayfish and shrimp (Böhm et al., 2001; Marder and Bucher, 2007; Dickinson et al., 2008; Hui et al., 2011; Tuszynski et al., 2015), species-dependent differences in the gastric mill and pyloric rhythms are readily recognizable. In a few cases, the apparently species-equivalent co-transmitting projection neurons have been studied in different crabs and lobsters. This includes comparison of MPN (*C. borealis*) and GN1/2 (GABA neuron 1/2: *Homarus gammarus*), as well as IVN (*C. borealis*, *P. interruptus*) and PS (*H. gammarus*, Russell and Hartline, 1981; Sigvardt and Mulloney, 1982; Claiborne and Selverston, 1984; Marder and Eisen, 1984; Meyrand et al., 2000; Christie et al., 2004; Kwiatkowski et al., 2013). The results of these studies highlight species-dependent similarities and differences in the co-transmitter content and function of the apparently same projection neuron. For example, the small molecule transmitter is unchanged (MPN, GN1/2: GABA; IVN, PS: histamine) but the peptide co-transmitter(s) differ(s) (Table 2). However, even when a co-transmitter was conserved across species, it did not always perform the same function, and in some cases the changed peptide co-transmitter did perform a comparable function.

### Co-transmission Consequences: Future Directions

With the continual development of stimulation and imaging techniques of ever-increasing resolution, the impact of co-transmission on circuit activity has blossomed to include many more model systems (Barker et al., 2016; Qiu et al., 2016; Granger et al., 2017; Nusbaum et al., 2017; Nässel, 2018). These recent studies have revealed conservation of mechanisms across species and circuits (e.g., convergent and divergent co-transmission; different temporal dynamics of ionotropic and metabotropic co-transmission; focal regulation of co-transmitter release), as well as diverse new ways in which activity is modified by co-transmission. It is already clear that the flexibility imparted to circuit output by co-transmission firmly places the parallel goal of determining the connectome for particular behaviors as a necessary but not sufficient foundation for understanding the neuronal basis of behavior (Bargmann, 2012; Bargmann and Marder, 2013; Meinertzhagen, 2018).

Despite the already evident diversity of mechanisms by which co-transmission alters circuit output, the future promises more surprises. For example, to date most co-transmission studies, in the STNS and elsewhere, have focused on the circuit response to single co-transmitter inputs/populations. However, it is likely that circuit operation *in vivo* receives parallel input from different neurons. This raises the issue of whether the consequences of parallel co-transmission will be evident from studying the impact of the individual components. Moreover, given that the impact of even individual co-transmitting inputs on circuit output is state-dependent (e.g., dependent on the physiological state of the target circuit, as well as the firing pattern and relative amounts of co-transmitters released by the inputs), each such study will need to be performed under rigorously defined conditions. Ultimately, such studies may require a blend of *in vitro* and *in vivo* recordings and manipulations to best establish both mechanism and behaviorally appropriate “state.”

There are also likely to be new functions revealed for co-transmitters. For example, in the *C. borealis* STG the co-release of the peptide proctolin from the projection neuron MCN1 does not directly influence any of the GMR generator neurons (Stein et al., 2007). However, a recent study suggests that MCN1-released proctolin may well indirectly influence GMR generation by slowing the enzymatic degradation of co-released CabTRP Ia. Specifically, [des-Arg^1^] proctolin was recently identified as a cleavage product of scorpion venom which effectively inhibits the endopeptidase neprilysin in arthropods (Duzzi et al., 2016). Neprilysin is likely the extracellular peptidase in the STG neuropil that cleaves and inactivates CabTRP Ia (Wood et al., 2000), and [des-Arg^1^] proctolin is the first cleavage product of proctolin in the STG (Coleman et al., 1994; Wood et al., 2000).

As already established in some systems, the release of different co-transmitters can be separately regulated and this regulation can occur focally, such as at particular axon terminals (DeLong et al., 2009a; Nusbaum et al., 2017). Under such conditions, the same neuron(s) can release different relative amounts of its co-transmitter complement from different release sites. Such compartmentalization further challenges investigators aiming to elucidate the cellular and synaptic mechanisms by which co-transmission affects neural signaling.

Understanding the impact of co-transmission, even on circuits composed of a small number of neurons, has benefitted from the use of computational models (DeLong et al., 2009b). As co-transmission studies scale up, both in terms of circuit size and the number of degrees of freedom made possible by co-transmission, hopes of attaining deep insight into the functional consequences of such events will be buoyed by the ever-increasing collaboration between experimentalists, theorists and modelers.

## Spinal Cord Modulation and Co-transmission

The spinal cord contains numerous transmitters in descending, sensory, and intraspinal systems (Hökfelt, 2009). These generally lack organizing principles, with the exception of 5-HT which Jacobs and Fornal (1993) proposed biases motor over sensory activity (Jacobs and Fornal, 1993). To illustrate the transmitter complexity of the spinal cord, consider the dorsal horn (this differs between regions and species, and thus this summary is not definitive; see Todd, 2010). Transmitter co-localization is common, as are ligand–receptor mismatches indicative of volume transmission.

Most nociceptive Aδ and C fibers terminate in laminae I–II, and mechanoreceptive Aβ-fibers in laminae III–VI. Laminae I-III contains densely packed neurons: most are local inhibitory interneurons, the remainder local excitatory or projection neurons (Todd, 2010). Glutamate is released from primary afferents and from local and descending neurons. Metabotropic glutamate receptors are concentrated on local interneurons in lamina II, while ionotropic glutamate receptors are found in all dorsal horn laminae and on primary afferent terminals. GABAergic neurons are found in laminae I-III. These neurons activate GABA_A_ and GABA_B_ receptors to regulate nociceptive and mechanosensory inputs. GABA receptor levels are reduced by peripheral nerve lesions, suggesting they are located on afferent terminals and GABA exerts its effects presynaptically. Glycine receptors are found in lamina 1 and II, but at greater levels in deeper laminae. Glycine receptor levels are not significantly affected by peripheral lesions, suggesting localization on dorsal horn neurons [glycine receptors may be present on some low threshold mechanosensory afferent terminals (Todd, 2010)]. Glycine and GABA can co-localize, and glycine and GABA_A_ receptors are found at many postsynaptic specializations in laminae I-III. ACh receptors are also present in lamina III-V, and a cholinergic plexus in lamina II-III receives inputs from unmyelinated and myelinated axons. Conventional transmitters can thus regulate specific afferent inputs presynaptically and/or postsynaptically.

The monoamines 5-HT and NA are found at all levels of the dorsal horn where they can act on dorsal horn neurons or afferent terminals. Both monoamines are predominantly released from descending brainstem neurons (these neurons provided key evidence of transmitter co-localization, see Hökfelt, 2009). For example, 5-HT is released from different raphe nuclei to affect sensory, motor and autonomic functions (Ghosh and Pearse, 2014). Rostral raphe neurons project to the dorsal horn and contain 5-HT and possibly GABA, while caudal raphe neurons project ventrally and co-localize 5-HT, glutamate, substance P, and TRH (Hökfelt et al., 2000). 5-HT is released synaptically in the ventral spinal cord, but paracrinally in the dorsal horn (Perrier and Cotel, 2015).

Neuropeptides are concentrated in laminae I and II [e.g., TRH, enkephalins, bombesin, substance P, vasoactive intestinal peptide (VIP), somatostatin, neurotensin, Cholecystokinin (CCK), neuropeptide Y (NPY), galanin], but also at deeper levels [e.g., substance P, enkephalins, somatostatin (Todd, 2010)]. Neuropeptides are found in descending or afferent neurons [(e.g., Galanin, CGRP SP, somatostatin, VIP, and CCK; peptides seem to be absent in Aβ fibers), and dorsal horn neurons (e.g., neurotensin and NPY)]. There are no absolute divisions in terms of where individual peptides are found, for example, substance P is found in Aδ and C afferents, in dorsal horn neurons, and in descending neurons (Jessell et al., 1979). Receptor localization is also diffuse: opiate receptor levels are reduced but not abolished following dorsal rhizotomy, suggesting they are located presynaptically on afferent terminals and postsynaptically on dorsal horn neurons. Peptides co-localize with amino acid transmitters: neurotensin, somatostatin and neurokinin B in glutamatergic neurons, galanin and NPY in GABAergic neurons, and others (e.g., enkephalins) in both glutamatergic and GABAergic neurons (Zhang et al., 1993; Xu et al., 2008; Sardella et al., 2011). Peptides also co-localize: met-enkephalin neurons contain tachykinins and somatostatin. As tachykinins and somatostatin do not co-localize there are probably distinct sub-populations of enkephalin-containing cells. CGRP co-localizes with substance P, somatostatin, or galanin. Galanin and substance P, and substance P and somatostatin co-localize, but galanin and somatostatin do not (Ju et al., 1987), again suggesting different sub-populations of peptidergic neurons.

This multiplicity of transmitters is a conserved feature, but is it necessary? Mammalian neurotransmitters serve diverse functions in bacteria and plants, and were presumably co-opted for neuronal signaling (Roshchina, 2010). The specific patterns of location and co-localization suggest functional relevance. This is supported by changes in transmitter systems with injury or disease. For example, galanin levels are up-regulated after dorsal root transection (Xu et al., 2008). Peptide receptor levels also change: inflammation increases delta opiod receptors in dorsal root ganglia and dorsal horn neurons to enhance endogenous analgesia, whereas substance P receptors are internalized (Merighi et al., 2011).

In principle one transmitter acting on multiple receptors could evoke excitation, inhibition, and modulation (Eccles, 1982). However, selecting these effects would be difficult if receptors were located at the same postsynaptic sites. Presynaptic regulation would also be difficult as autoreceptor activation would result whenever the transmitter was released. Spatially separating synapses/receptors could segregate effects, but this places demands on spinal cord size and organization. Differential effects could occur if receptors serving different functions had different thresholds, but this would be limited. Consider presynaptic regulation of transmitter release from Neuron1 by Neuron2. A high threshold receptor on Neuron1 terminals would prevent its activation when Neuron1 released transmitter, but this would demand greater release from Neuron2 to activate the receptor to evoke presynaptic regulation. Any autoreceptors on Neuron2 would then need an even higher threshold to prevent their activation, leading to ever increasing thresholds and release levels as system complexity increased. Threshold level regulation would also limit signaling distances, reducing the opportunity for volume transmission (Fuxe et al., 2010). As a specific example consider the activity-dependent potentiation of nociceptive transmission (“wind-up”) in the dorsal horn (Dubner and Ruda, 1992). Substance P released from nociceptive afferents potentiates NMDA responses to postsynaptically increase nociceptive responses: this can be reduced by opioid mediated pre- or postsynaptic inhibition. For wind-up to occur using only glutamate, increased glutamate release following nociceptor activation could activate higher threshold NMDA-receptors to trigger Ca^2+^-dependent 2nd messenger pathways that potentiate nociceptive signaling. Descending glutamatergic inputs could act on mGluRs to pre- or postsynaptically inhibit responses, but to prevent mGluR activation by afferent glutamate release would require the higher activation threshold or spatial separation outlined above. Multiple transmitters are clearly advantageous.

### Studying Spinal Cord Modulation

Analyses of spinal cord motor outputs and their modulation have typically used fictive locomotion (pharmacologically or electrically-evoked activity recorded from ventral roots in isolated spinal cords). However, assumptions that fictive activity matches normal locomotion has been questioned on experimental and conceptual grounds see Ayers et al. (1983); McClellan (1990); Musienko et al. (2012) and Parker and Srivastava (2013) and references therein. In lamprey, modulation of fictive and actual locomotion differs (Kemnitz et al., 1995; Becker and Parker, 2015). We thus need to ensure that fictive effects give physiologically-relevant information.

Given the difficulty of specifically activating modulatory systems, endogenous release is typically studied by blocking uptake. However, this does not determine how or when release occurs to evoke specific effects. Exogenous application allows known concentrations of one or more transmitters to be examined at specific times, but as normal spatial and temporal signals are not mimicked, effects may not be physiologically-relevant [exogenous and endogenous 5-HT have different effects on locomotor outputs and motor neuron excitability (Perrier and Cotel, 2015)]. Given the number of transmitters and possible combinations, we cannot examine co-transmission by analyzing all exogenous interactions individually, but have to try to determine the rules underlying these interactions (Furness et al., 1989).

Understanding functional effects requires a characterized network where convergent and divergent modulation of identified circuit components can be characterized (Harris-Warrick and Johnson, 2010). Gaps in even the simplest spinal cord locomotor networks (see Parker, 2006, 2010) mean that caution is needed in claiming understanding of how modulators evoke their effects. The effects of substance P and 5-HT in the lamprey, a lower vertebrate spinal cord model, will be used to illustrate limits to our understanding of spinal cord neuromodulation and modulator interactions.

### Spinal Cord Modulation and Interactions in Lamprey

5-HT slows the frequency of fictive locomotion in lamprey (Harris-Warrick and Cohen, 1985), and reduces the slow afterhyperpolarization (sAHP) following an action potential (Van Dongen et al., 1986a). These effects were causally linked (Grillner et al., 1995): sAHP summation terminates spiking; the 5-HT-mediated reduction of the sAHP thus prolongs spiking; this will delay locomotor burst termination, thus slowing the locomotor burst frequency. This scheme was supported by a computer simulation (Hellgren et al., 1992), albeit requiring *ad hoc* adjustments of the network architecture (see Parker, 2006). In addition, the claimed causal link rested on two assumptions: that 5-HT affected the sAHP in appropriate network neurons; and that the sAHP reduction was the only effect of 5-HT. The first assumption remains uncertain (Parker, 2006), while the second was unlikely given divergent modulator effects and that 5-HT could hyperpolarize spinal cord neurons (Harris-Warrick and Cohen, 1985). 5-HT modulation of inhibitory and excitatory synaptic inputs to motor neurons was subsequently shown (Parker, 2006). 5-HT’s net synaptic effect is reduced excitation, which can slow simulated and fictive network activity (Brodin et al., 1985). Two simulations (Hellgren et al., 1992; Kozlov et al., 2001) thus show the same effect using different cellular assumptions. Several divergent mechanisms individually or in combination could thus underlie the network effects of 5-HT, but their relative causal influences remaining unknown.

Substance P evokes a long-term increase in the frequency and improvement in the regularity of fictive activity in the lamprey (Parker et al., 1998) and neonatal rat (Barthe and Clarac, 1997). In lamprey, the long-term burst frequency effect is NMDA-, PKC-, and protein synthesis-dependent: the burst regularity effect is protein kinase A-dependent but NMDA- and protein synthesis-independent (Parker et al., 1998). Substance P has varied cellular and synaptic effects (Parker et al., 1997; Parker, 2006), and thus like 5-HT conforms to the general principle of divergent cell and synapse-specific modulation (Harris-Warrick et al., 1998). While the induction of the long-term burst frequency effect depends on postsynaptic NMDA receptor potentiation (Parker et al., 1998), its maintenance and the mechanisms underlying the improved burst regularity are unknown (Bevan and Parker, 2004; Parker and Bevan, 2007). As with 5-HT, the complexity of even this simpler spinal cord system means that the network effects of substance P cannot be causally reduced to cellular mechanisms (Parker and Grillner, 2000).

5-HT and substance P also illustrate modulator interactions. Both are found in a ventromedial spinal cord plexus where 5-HT and dopamine co-localize: tachykinins co-localize in a subset of 5-HT/dopamine cells (Van Dongen et al., 1986b). Exogenous 5-HT application blocks the presynaptic and postsynaptic effects of substance P on glutamatergic transmission and the long-term network modulation. Dopamine does not interact with substance P, but removes the 5-HT-mediated block of the presynaptic substance P effect to allow a short-term increase in glutamatergic synaptic transmission and the network burst frequency. As dopamine does not remove the 5-HT-mediated block of the NMDA receptor potentiation by substance P, the long-term network effect remains blocked (Svensson et al., 2001).

Other neuropeptide interactions also occur in lamprey. Peptides can modulate various reflex responses (Ullström et al., 1999). CCK and peptide YY (PYY) are co-transmitters in descending glutamatergic brainstem neurons, and CGRP and NPY co-transmitters in glutamatergic sensory neurons. All reduce the amplitude of low-frequency-evoked reticulospinal inputs (again when applied exogenously), but the sensory peptide effects are blocked by the brainstem peptides (Parker, 2000).

### Spinal Cord Injury

Neuromodulation of remaining sensory or motor networks offers the potential for interventions after spinal cord injury (SCI; Rossignol and Frigon, 2011). However, despite extensive effort there is still little indication of an optimal pharmacological approach. This reflects the difficulty of understanding how even single modulators evoke their effects (see above), which makes rational targeted interventions difficult. Time after injury and the extent of the lesion also need to be considered, as these can evoke state-dependent variability that alter drug or transmitter effects (Rossignol and Frigon, 2011; Parker, 2015). As systemically-applied drugs and certain transmitters will work through volume transmission, changes in the extracellular space in the acute or chronic phases after SCI could change the spatial and temporal characteristics of endogenous or exogenous interactions.

5-HT is the best studied transmitter/modulator after SCI (Antri et al., 2003; Musienko et al., 2011). 5-HT is released from different raphe nuclei to affect sensory, motor and autonomic functions (Ghosh and Pearse, 2014). Intraspinal 5-HT systems in lower vertebrates that co-localize other amines and peptides (Schotland et al., 1996) can also appear after SCI in mammals (Ghosh and Pearse, 2014). There are over 30 5-HT receptor subtypes located presynaptically, postsynaptically, or extrasynaptically in the spinal cord (Jordan et al., 2008; Cotel et al., 2013). 5-HT usually slows locomotor activity, but its effects differ in different systems (Sillar et al., 1998), and on whether fictive or actual locomotion is examined (Kemnitz et al., 1995; Becker and Parker, 2015), and 5-HT can excite or inhibit motor neurons (Perrier and Cotel, 2015). This diversity presumably reflects the net effect of activating multiple 5-HT receptors.

Damage to serotonergic pathways has been implicated in various aspects of SCI, including paralysis, spasticity, and neuropathic pain. Descending 5-HT and noradrenergic inputs presynaptically inhibit proprioceptive and nociceptive afferents and interneuronal pathways through G_i_protein-coupled receptors e.g., 5-HT_1A_, 5-HT_1B_, 5-HT_1D_, and α2-adrenergic receptors (Nardone et al., 2015). Damage to these pathways thus disinhibits sensory inputs leading to spasticity (Li et al., 2004). Motor neuron hyperexcitability also contributes to spasticity in rats and humans (Li et al., 2004; Norton et al., 2008). 5-HT and NA normally facilitate motor neuron function through 5-HT_2_ and α1-mediated persistent sodium and calcium currents (Perrier et al., 2003). After SCI motor neurons are initially unexcitable (Li et al., 2004; Heckmann et al., 2005), leading to arreflexia and spinal shock, but large persistent calcium and sodium currents subsequently develop that increase motor neuron excitability. This can occur through constitutive activation of 5-HT_2_ and α1-receptors in motor neurons (Harvey et al., 2006; Murray et al., 2010) or denervation supersensitivity of 5-HT receptors and downregulation of 5-HT uptake (Husch et al., 2012). Interestingly, contusion injuries that spare some serotonergic projections lack these effects despite 5-HT receptors being upregulated (Hayashi et al., 2010).

Various aminergic receptors are upregulated after SCI (Rossignol et al., 2001). While the simplest interpretation is that these receptors evoke the same effects in lesioned and unlesioned spinal cords, this may not be the case. Somatostatin, GABA and 5-HT modulation differ after SCI in lamprey (Svensson et al., 2013; Becker and Parker, 2015), and aminergic and glutamatergic effects differ after SCI in mammals (Giroux et al., 1998, 2003). The potential differences in transmitter effects after SCI need to be understood if targeted pharmacological interventions are to be effective.

We lack sufficient insight into modulator effects and their interactions after SCI. Endogenously released neuromodulators can evoke diverse sensory and motor effects through wired and volume transmission. We need to know if and how these effects differ after SCI. Concentration-dependent effects and production of metabolites with different effects along volume transmission pathways could produce varied temporal and spatial-dependent signals from a single transmitter, all of which could be altered by injury-induced changes in the extracellular space. Interestingly, removal of proteoglycans, intended to promote axonal regeneration across lesions (Muir et al., 2017), will also alter the extracellular space, and it should be considered if any functional effects seen with proteoglycan removal reflects changes in volume transmission rather than regeneration. Finally, changes in functional properties, which are ubiquitous below lesion sites could evoke state-dependent changes. It seems unlikely that modulation after SCI could be reduced to a single variable. Rational interventions require greater insight into spinal cord modulation and co-transmission before and after SCI.

## Hypothalamic Co-release

While model systems allow detailed analyses of neuronal and circuit-level consequences of co-transmission, we ultimately have to understand these effects in more complex systems. A recent approach to investigating the synaptic consequences of co-transmission is optogenetic circuit mapping strategies, where light-sensitive opsins are virally-expressed in neurochemically-distinct neurons that are subsequently activated *ex vivo* in the presence and absence of pharmacological blocking agents. This significant development allows endogenous transmitter release and post-synaptic activity to be examined, instead of relying on exogenous application (Qiu et al., 2016). This has shown that whether the co-release of fast and relatively slow-acting transmitters act co-operatively, in an additive fashion, or independently of each other to influence the output of post-synaptic targets seems to differ amongst various systems.

### Antagonistic Inputs Onto Hypothalamic Arousal Circuits Regulate Wakefulness and Sleep

**Figure 6:**
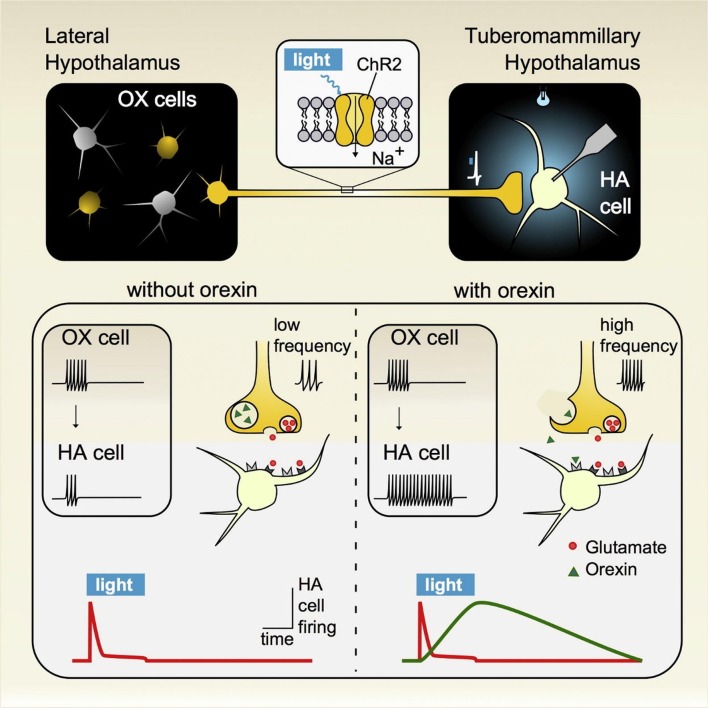
Activation of hypothalamic orexin neurons that impact wakefulness-promoting histamine neurons generates distinct signature excitatory responses resulting from the co-release of glutamate and the neuropeptide hypocretin/orexin (HO). (Top panel) A cre-recombinase approach was used to express the light-activating excitatory protein channelrhodopsin (ChR2) in orexin neurons in orexin-cre transgenic mice. Brief pulses of light were sufficient to evoke transmitter release from orexin terminals that then impacted postsynaptic histamine neurons, which were identified by intrinsic signature currents and *post hoc* immunoprocessing for histamine decarboxylase reactivity. (Bottom panel) Low frequency stimulation led to the synaptic release of glutamate only while under high firing regimes both glutamate and orexin were co-released. In response to this co-transmission, the evoked responses measured on histamine neurons were sequential, where glutamate release and its corresponding postsynaptic excitation was fast and transient, lasting only while orexin neurons were active while orexin independently excited histamine neurons in a delayed fashion and whose response outlasted the stimulation duration. These results indicate that glutamate/orexin cotransmission may translate distinct features of orexin activity into parallel, nonredundant signals for regulating distinct circuits important for generating appropriate levels of arousal for maintaining wakefulness. Figure adapted with permission from Schöne et al. (2014) and the publisher.

Using an optogenetic approach in acute hypothalamic slices, Schöne et al. (2014) demonstrated that hypocretin/orexin (HO) and glutamate released from HO neurons act independently of each other to influence postsynaptic histamine neuronal targets on different timescales (Schöne et al., 2014). This circuit plays an important role in arousal and maintaining wakefulness, highlighting the importance of this projection in sleep-related disorders (Huang et al., 2001). HO-expressing neurons exclusively reside in the hypothalamus and project ubiquitously throughout the brain (Tsunematsu and Yamanaka, 2012). Shortly after their discovery in the late 1990s, genetic knockout studies showed that HO plays an important role in arousal as it relates to wakefulness and for generating appropriate reward-seeking behavior specifically in regards to maintaining energy balance (de Lecea et al., 2006; Sakurai, 2007). With the development of novel circuit mapping strategies, experiments began to reveal not only the HO pathways involved in mediating these processes, but also the underlying synaptic mechanisms. *In vivo* optogenetic circuit analyses demonstrated that HO inputs to the tuberomammillary nucleus (TMN), where histamine neurons reside, are critical for maintaining wakefulness (Huang et al., 2001). Moreover, *ex vivo* interrogation of this circuit has shed light on the synaptic mechanisms through which HO acts on the histamine arousal circuit. Schöne et al. (2014; Figure 6) demonstrated that HO neurons excite histamine neurons through both glutamate and HO release that together cooperate in facilitating histamine neuron firing. But instead of having an additive effect, light-evoked glutamate and HO released from HO-expressing neurons act independently of each other, as pharmacological blockade of each receptor type selectively affected that receptor without altering responses to the co-transmitter. These findings indicate that co-transmission of glutamate and HO on spike responses could co-exist in the same postsynaptic cell and non-redundantly activate histamine arousal circuits for maintaining wakefulness. At low firing frequencies HO neurons generate a glutamate-mediated tonic excitatory tone in histamine neurons, while at higher firing frequencies HO peptides are released and can sustain the excitatory tone of histamine neurons long after the HO-expressing neurons fall silent. These results are consistent with, and expand on previous studies demonstrating that HO activity promotes awakening in a frequency-dependent manner (Adamantidis et al., 2007), while loss of HO neurons results in narcolepsy (Thannickal et al., 2000; Hara et al., 2001). Lack of HO peptides or HO type-2 G protein coupled receptors (HOR2), the subtype expressed by histamine neurons, results in a similar phenotype, highlighting the clinical significance of HO neurons (Chemelli et al., 1999; Lin et al., 1999) and their projection onto histamine neurons.

The hypothalamic HO wakefulness system is complemented by the sleep-promoting melanin-concentrating hormone (MCH) system. Hypothalamic MCH neurons send widespread central projections to support a diverse set of neural processes (Girardi et al., 2018). Locally in the hypothalamus, however, they play an important facilitating role in NREM and REM sleep (Ferreira et al., 2017). Similar to HO neurons, their axons synapse onto TMN histamine where they reduce histamine neuron activity through GABAergic synaptic transmission (Jego et al., 2013). Moreover, high frequency photo-stimulation results in an increase in the frequency of inhibitory IPSPs while the overall amplitude remained the same. Repeating the stimulation protocol in MCH receptor 1 knockout mice did not result in an increase in IPSP frequency leading the authors to conclude that the evoked IPSP potentiation was likely due to synaptically-released MCH that acts presynaptically on MCH terminals to increase GABA release. The net effect is a greater suppression of histamine neuronal activity with increases in MCH activity. A similar presynaptic mechanism of action has been documented in HO neurons where elevated HO activity increased GABAergic tone in MCH neurons (Apergis-Schoute et al., 2015). In contrast to HO’s ability to enhance histamine neuronal output, thereby promoting wakefulness, the combined inhibitory impact of GABA and MCH co-transmission onto histamine neurons is thought to reduce histamine release and as a result contribute to the sleep-promoting feature of MCH neuronal transmission.

### Hypothalamic Cell Populations Important for Maintaining Energy Homeostasis Are Functionally Connected

Distinct hypothalamic regions are involved in food-seeking behavior related to maintaining energy balance. The hypothalamic melanocortin system in particular has received much attention, as the melanocortin receptor 4 (MC4R) in the paraventricular nucleus of the hypothalamus (PVH) can differentially impact food intake, see Krashes et al. (2016). Importantly, this depends on projections from the arcuate nucleus, another hypothalamic brain region known to be important in regulating food intake relating to energy homeostasis. Arcuate agouti-related protein expressing neurons (AgRP) and pro-opiomelanocortin (POMC)-containing neurons have opposing actions on PVH neurons that can increase or decrease food intake (Sternson, 2013), implicating these cell populations in hunger and satiety states, respectively. Zhang and van den Pol (2016) examined a third arcuate cell population, one that contains the dopamine precursor enzyme tyrosine hydroxylase (TH), and showed that short-term optogenetic activation of arcuate-TH neurons transiently increased food intake in transgenic TH-cre mice, while long-term disruption in arcuate-TH activity reduced their body weight measured over the course of months (Zhang and van den Pol, 2016). The increased food intake can be partially attributed to synaptic influences of arcuate-TH neurons on PVH neurons, as photo-stimulating their axons *ex vivo* led to inhibition of satiety-signaling PVH neurons. Interestingly, at high firing frequencies arcuate-TH neurons co-released GABA and dopamine resulting in an inhibition of PVH neurons. In contrast to co-transmission of HO and glutamate from HO neurons, GABA and dopamine release from arcuate-TH neurons had additive post-synaptic inhibitory effects. Investigation of whether synaptic inputs from arcuate-TH to AgRP or POMC can facilitate food intake by respectively exciting or inhibiting these arcuate populations found that photo-activating arcuate-TH terminals resulted in a GABA-mediated inhibition of POMC neurons while no synaptic transmission was measured on AgRP neurons. In subsequent *ex vivo* experiments, bath-applied dopamine inhibited POMC neurons and excited AgRP neurons but whether or not these opposing effects were due to synaptic dopamine release from arcuate-TH neurons was not directly tested. When neuronal excitability was monitored using cFos as a molecular marker of activity, *ex vivo* whole-cell recording techniques showed that a state of hunger markedly increased arcuate-TH activity. Together, these results are consistent with the premise that by acting on hypothalamic circuits that regulate appetite, arcuate-TH neurons can drive hunger-related food intake. In the arcuate to PVH pathway, these neurons can synaptically influence post-synaptic targets that regulate appetite through GABA and dopamine co-release. Although activation and inhibition of arcuate-TH neurons has revealed an important role for these cells in generating feeding behavior, the exact contribution of GABA and dopamine co-transmission from arcuate-TH neurons for normal food intake is yet unresolved.

### Functionally Connected Hypothalamic Circuits Are Important for Balancing Homeostatic Processes Critical for Survival

Through direct and indirect synaptic influences on one another, hypothalamic circuits with complementary appetite, sleep/wake, and other important homeostatic functions are thought to be under the influence of various sensory and physiological cues. When the system is imbalanced these signals, through synaptic processes within hypothalamic circuits, can add weight to complementary circuits for shifting the system towards homeostatic responses. This constant interplay between opposing systems ultimately changes the animal’s state and defines which behavior is appropriate for survival. It is of particular interest that a number of neural disorders that involve disturbances in elementary drives critical for survival involve disruptions in hypothalamic circuits, in particular ones containing specialized neuropeptides (Krude et al., 1998; Yeo et al., 1998; Thannickal et al., 2000). Disruptions in their synthesis or transmission have often been linked to specific disorders, but what is not clear is whether or not these neuropeptide systems co-release other transmitters and if so how they interact in generating appropriate behavior. In light of this, a better understanding on the relationship between transmitter release by neurochemically-distinct hypothalamic cell populations and their post-synaptic impact will shed light on the synaptic mechanisms that regulate homeostatic processes important for survival.

## Rodent Neocortex and Basal Ganglia

### The Neocortical Neuronal Circuit

**Figure 7:**
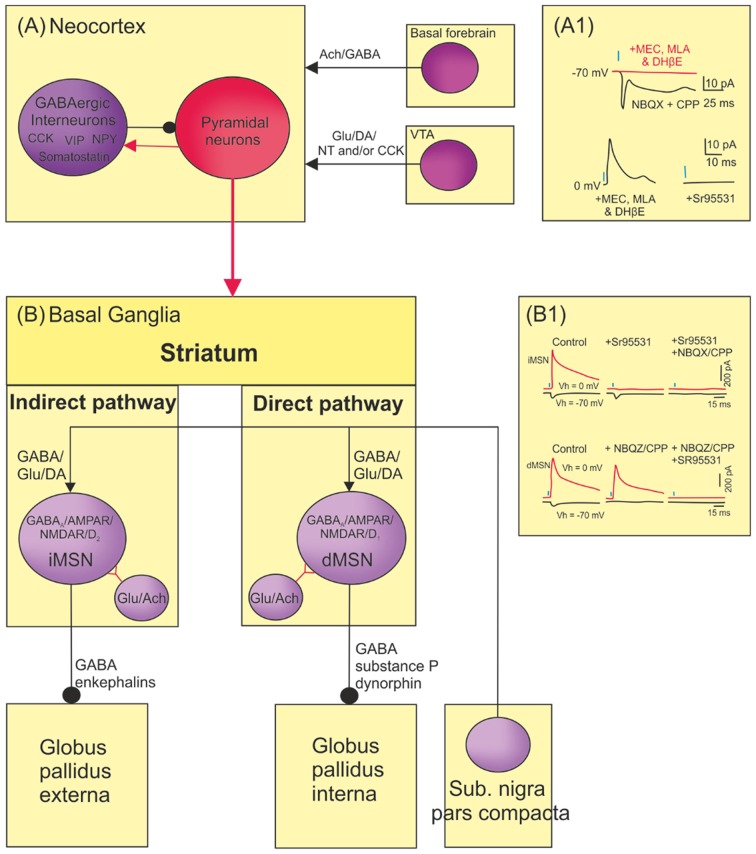
Co-transmission in higher brain circuits (neocortex and basal ganglia). **(A)** In the cortical column GABAergic interneurons co-localize a range of different neuropeptides that can contribute to the tuning of activity of the cortical column. The neocortex also receives innervation from subcortical nuclei that can influence the tuning of cortical network activity. The projection from ventral tegmental area (VTA) co-localize glutamate, dopamine and/or the neuropeptides neurotensin and CCK. The cholinergic projections from forebrain neurons co-release GABA and thus activates nAchR and GABA_A_ receptors in cortical neurons. **(A1)**. Traces showing nicotinergic receptor and GABA_A_ receptor antagonism of optogenetically generated synaptic potentials. **(B)** In the basal ganglia, GABAergic medium spiny neurons (MSN) in the direct pathway co-localize substance P and dynorphins, while MSN in the indirect pathway co-localized enkephalins. Cholinergic interneurons in striatum co-localize glutamate and have excitatory action onto MSN. Dopaminergic neurons from substantia nigra pars compacta also co-localize and co-release GABA and glutamate. This input to striatum will thus activate GABA_A_, AMPA, NMDA, and D_1_ (dMSN) or D_2_ (iMSN) receptors on MSN in the dorsal striatum. **(B1)** Traces showing that optogenetic activation of dopaminergic neurons activates GABA_A_ receptors and glutamatergic AMPA and NMDA receptors. Adapted from Lobo (2009), El Mestikawy et al. (2011), Higley et al. (2011), Tritsch et al. (2012, 2016), Kabanova et al. (2015), Saunders et al. (2015a,b), Morales and Margolis (2017).

In mammals, the neocortical circuitry is relatively well-described (Markram et al., 2015) and suitable for investigations into co-transmission and neurotransmitter interactions. The neocortex is richly innervated by peptidergic interneurons containing tachykinins, enkephalins, somatostatin, NPY, VIP and CCK. The anatomical basis for these peptides is to some extent known and many of them have been localized to GABAergic interneurons (e.g., basket and Martinotti cells; Figure 7A). However, relatively little is known about their modulatory effects, interactions, or co-release (Markram et al., 2004; Rudy et al., 2011). Neuromodulation of short-term synaptic plasticity (metaplasticity) is an important factor for the regulation and operation of oscillatory hard-wired neuronal networks, which could contribute to fine-tuning the neocortical activity (Parker and Grillner, 1999; Abraham, 2008). Since the modulatory effects of amines and neuropeptides are long lasting, they can interact, even if their release is both temporally and spatially separate, a phenomenon known as metamodulation (Katz and Edwards, 1999; Svensson et al., 2001).

The neocortex is also innervated by aminergic and cholinergic projections originating from the midbrain that co-localize other transmitters such as glutamate and GABA, as well as neuropeptides (Kabanova et al., 2015; Root et al., 2016; Schultz, 2016a; Morales and Margolis, 2017), conforming to the general principle of amino acid, aminergic, and peptidergic co-localization outlined above. Dopaminergic inputs from VTA to the cortex co-localize glutamate, as well as the neuropeptides neurotensin and/or CCK (Figure 6A of Kabanova et al., 2015; Morales and Margolis, 2017). The co-localization of dopamine and glutamate is common and found in systems from lamprey to the human brain (Root et al., 2016). The midbrain dopaminergic system generates a reward signal critical for motor skill learning (Hosp et al., 2011; Kunori et al., 2014; Rioult-Pedotti et al., 2015; Schultz, 2016a), and dopaminergic modulation has also been implicated in working memory in the prefrontal cortex (Goldman-Rakic, 1995). By combining optogenetics with electrophysiological patch-clamp techniques, VTA projections have been found to make excitatory synapses onto prefrontal glutamatergic and GABAergic neurons (Kabanova et al., 2015; Pérez-López et al., 2018). Dopamine could promote memory formation by generating a reward signal that makes the cortical network adaptable and plastic, changing neuronal circuit activity to generate new motor skills (Goldman-Rakic, 1995; Hosp et al., 2011; Kawai et al., 2015; Schultz, 2016a). Dopaminergic neurons could reduce errors by generating instructive feedback signals (Hosp et al., 2011; Kawai et al., 2015).

Cholinergic neurons from the basal forebrain co-localize GABA (Saunders et al., 2015a,b; Granger et al., 2016) and thus activate nicotinic and GABA_A_ receptors in cortical neurons (Figure 7A1). The role of ACh/GABA co-transmission is not well understood, but may contribute to motor memory formation in the primary motor cortex (Conner et al., 2010; Saunders et al., 2015a,b; Tritsch et al., 2016). Interestingly, this cholinergic/GABAergic pathway contributes to rehabilitation of motor functions after cortical injuries, and degenerates in Alzheimer’s disease, making the functional role of this co-transmission of clinical interest (Tuszynski et al., 2015; Wang et al., 2016). However, the behavioral consequences of the modulatory interactions resulting from their potential co-release are poorly understood (El Mestikawy et al., 2011; Saunders et al., 2015a,b).

### The Basal Ganglia and Substantia Nigra Pars Compacta

GABAergic medium spiny neurons (MSN) in the basal ganglia can be divided into two functional populations, the direct and indirect pathways, used for the initiation and termination or inhibition of movements, respectively (Nelson and Kreitzer, 2014). GABAergic neurons of the indirect pathway co-localize the neuropeptide enkephalin and those of the direct pathway co-localize the neuropeptides substance P and dynorphin (Figure 7B). Excitatory interneurons in the striatum also co-localize ACh and glutamate to excite the MSN, as well as fast spiking interneurons that mediate a disynaptic GABAergic inhibition onto MSNs (Higley et al., 2011; Nelson et al., 2014; Figure 7B).

Dopaminergic inputs to striatum from the substantia nigra pars compacta are important for reward and the normal function of striatal neuronal circuitry (Schultz, 2016b). They also co-localize GABA and glutamate (Figures 7B,B1; Tritsch et al., 2012, 2016; Stensrud et al., 2014; Berrios et al., 2016; Chuhma et al., 2017). Dopamine activates D_1_ receptors in the direct pathway but D_2_ receptors in the indirect pathway. Glutamate activates AMPA and NMDA receptors and GABA acts on GABA_A_ receptors (Figure 7B1; Tritsch et al., 2012). GABA is released from the same vesicles as dopamine and is transported into the vesicle by vesicular monoamine transporter (VMAT2). This complex synaptic arrangement combined with multiple receptors could give rise to new and non-linear modulatory actions and thus flexible regulation of MSN activity. Since dopaminergic projections degenerate in Parkinson’s disease and MSNs in the indirect pathway degenerate in Huntington’s chorea, it will be important to understand the functional role of co-transmission in these circuits to develop effective treatments, by either improving the efficacy of existing treatments (e.g., L-Dopa), suggesting new potential pharmacological strategies, or facilitating the effects of other approaches (e.g., as an adjunct to deep brain stimulation).

Furthermore, dopaminergic projections from the VTA to nucleus accumbens co-localize dopamine and glutamate and generate a reward signal. This input is implicated in drug addiction conditions (Morales and Margolis, 2017). However, the two different transmitters are segregated and are localized in both different vesicles and synaptic specializations (Zhang et al., 2015).

## Conclusions

Co-transmission is clearly a ubiquitous feature across systems of differing complexity and serving different functions. This makes determining its general principles important to our basic understanding of nervous system signaling mechanisms. As co-transmission is found in central circuits involved in disorders like Parkinson’s disease and Huntington’s chorea, understanding these principles could potentially improve existing pharmacological treatments or identify new ones. However, our understanding of the functional effects of co-localized neurotransmitters in higher functions is still in its infancy.

It seems unlikely that any normal or pathological function can be reduced to a single transmitter system. Current pharmacological approaches for neurological disorders, including SCI, have limited efficacy at best. This should be reflected in more caution in the claims for treatments and for interventions in normal function (e.g., cognitive enhancement; Sahakian et al., 2015). There could be many reasons for this limited efficacy, but the most obvious is that pharmacological approaches do not mimic the normal endogenous release of (co)transmitters or neurotransmitter interactions, and thus the normal chemical environment of the relevant circuits. As shown in the more accessible simpler systems discussed here (*Aplysia*, crustacean STNS, lamprey), exogenous application does not necessarily reflect normal functional effects. Endogenous release reflects specific patterns of activity that differentially release multiple transmitters that have multiple single and interactive effects on multiple receptors. While exogenous application was a useful and necessary simplification, it essentially “averages” effects across receptors and regions. We need to become more sophisticated in our approaches. The best current approach is optogenetic activation of modulatory systems, as illustrated by studies performed in hypothalamic circuits (see above). However, we need to ensure that the elegance of optogenetic approaches does not blind us to the requirement of ensuring that we are stimulating the appropriate neurons in a physiologically-relevant manner if the studies are to have optimal efficacy (e.g., Arrigoni and Saper, 2014). Given sufficient temporal precision, we may be able to optogenetically activate neurons to selectively release co-localized amines or neuropeptides to investigate their physiological and behavioral effects. Discrete modulatory systems can be localized in discrete regions (e.g., raphe nuclei and locus coeruleus), allowing optogenetic activation of these systems to be done with some precision (Miyazaki et al., 2014). In addition, optogenetically regulating intracellular processes (e.g., vesicle filling; Rost et al., 2015) could enable manipulation of subsets of co-localized transmitter vesicles to investigate the effects of intrinsic co-release during natural behaviors. This would allow the analysis of intrinsic effects to move beyond the traditional approach of blocking uptake, which provides little insight except to say that transmitters are released somehow, under some conditions, to evoke some effect, and provides an adjunct to studies that use optogenetic activation or inhibition of neurons. As useful as the latter approach is, it does not tell us about the natural release or effects of co-localized transmitters. Optogenetic manipulation of endogenous co-release could thus significantly advance out understanding of behavioral effects, arguably the major open question in studies of co-transmission.

Much of our basic insight into the mechanisms and functional relevance of co-transmission and transmitter interactions has come from invertebrate and lower vertebrate model systems or the peripheral nervous system (e.g., autonomic purinergic signaling). The information obtained in these systems provides a basis for understanding effects in more complex circuits, and highlights the utility of fundamental research in model systems. Insofar as general principles of co-localization and co-transmission are still lacking, these classical systems are likely to continue to provide important insights.

Elucidating co-transmitter function will continue to benefit from analyses in genetically-tractable model systems like *Caenorhabditis elegans* and *Drosophila melanogaster*. For example, in *C. elegans*, the modulation of various behaviors [e.g., egg laying (Chen Y. Y. et al., 2017); aggregation (Chen C. et al., 2017); aversive behavior (Mills et al., 2012; Clark et al., 2018] in response to specific conditions [e.g., food deprivation (Bhattacharya et al., 2014) or oxygen levels (Chen C. et al., 2017)] has been examined using anatomical, genetic, pharmacological, imaging and optogenetic approaches. These studies have included the effects amine and neuropeptide release and interactions (Mills et al., 2012; Ghosh et al., 2016), for example, through the co-release of different subsets of peptide co-transmitters from a single sensory neuron (Clark et al., 2018). As in mammals, neuropeptides form a major transmitter group in *C. elegans* (Van Bael et al., 2018a,b). There are approximately 119 neuropeptide genes, the products of which undergo posttranslational modifications to generate mature neuropeptides. These peptides form three classes: FMRFamide-like peptides (flps); insulin-like peptides (ins); and the largest group, neuropeptide-like proteins (nlps) that lack sequence similarity to FMRFamide or insulin. In common with other systems, these neuropeptides are stored in DCVs that can be released synaptically or extrasynaptically (see Janssen et al., 2010). Neuropeptides modulate at least several different *C. elegans* behaviors [e.g., FMRFamide promotes solitary over social feeding (de Bono and Bargmann, 1998; Leinwand and Chalasani, 2013)], while the nlp-12 gene influences food seeking behaviors (Bhattacharya et al., 2014; Chen C. et al., 2017; Chen Y. Y. et al., 2017; Iannacone et al., 2017; Stern et al., 2017; Buntschuh et al., 2018; Oranth et al., 2018). *Drosophila* has also provided important insights. For example, feeding reflects a hierarchical sequence of behaviors, including foraging and consummation, which in turn reflects the activation of various transmitter systems (see Ignell et al., 2009; Kahsai et al., 2010; Root et al., 2011; Choi et al., 2012; Kapan et al., 2012; Wang, 2012; Barnstedt et al., 2016; Kim et al., 2017; Nässel, 2018). Starved flies show facilitated synaptic outputs from Or42b olfactory receptor neurons (ORN) mediated by neuropeptide F (NPF), a peptide structurally and functionally related to mammalian NPY, but a tachykinin-mediated reduction of activity from Or85a ORNs: these two ORNs mediate odor-guided attraction and repulsion, respectively, and thus their net effect is attraction towards food. Neuromodulatory cascades also co-ordinate gustatory responses. The gustatory receptor neuron (GRN) Gr5a detects sugars while the Gr66a GRN detects bitter tastes. Starvation increases synaptic outputs from Gr5a through a NPF-mediated increase in the activity of the dopaminergic neuron TH-VUM. Starvation also causes the release of adipokinetic hormone (AKH) which increases the activity of NPF-releasing neurosecretory cells: these activate GABAergic neurons to reduce the activity of octopamine and tyramine-containing neurons, a sequence that ultimately reduces Gr66a activity. The net effect is increased attraction and reduced aversion to food cues. Starved flies also increase locomotor activity through AKH-mediated activation of octopaminergic neurons in the subesophageal zone (insulin inhibits these cells to signal satiety). Other neuromodulators (e.g., allatostatin-A, corazonin, drosulfakinin, and serotonin) also regulate feeding (see Kim et al., 2017 and references therein). These provide the opportunity to examine co-ordinated neuromodulator release and interactions. While electrophysiological analyses cannot typically be performed in *C. elegans*, and are less tractable in *Drosophila* than in the more accessible invertebrate systems such as *Aplysia* and the decapod crustacean STNS, activity can be tracked *via* Ca^2+^ imaging while molecular genetic approaches allow modulatory systems and their targets to be manipulated to investigate how co-localized transmitters and their interactions influence behaviors.

### General Principles

The chemical organization of synaptic terminals, where SSVs containing amino acids and DCVs containing amines and peptides are located at different sites in the terminal and are released by different Ca^2+^-dependent signals that reflect firing rates and patterns, appears to be a general feature of co-localization and co-transmission across systems. While specific releasing-stimuli can differ between neurons, lower firing frequencies tend to rapidly release SSVs, while higher frequency or burst firing elicits the relatively slow release of DCVs.

Flexibility is the usual reason given for the presence of multiple signaling molecules, each of which can elicit a different neuronal or circuit output, allowing hard-wired connectomes to generate a considerable diversity of outputs. This is a well-established general principle across systems.

Divergent and convergent signaling of modulators is also a highly conserved general principle conserved from simpler- to mammalian systems. Divergence is facilitated by the volume transmission of amines and especially peptides that allows a spatial field of effects from a point of release.

### Aspects to Be Addressed

While SSV release has been studied extensively, we still lack insight into the mode and mechanisms of DCV release (Xia et al., 2009; Bulgari et al., 2018). To understand the regulation and role of co-release we need to understand the specific release parameters of the different classes of vesicles. These parameters are unlikely to be linear, each type of vesicle probably having specific activity-dependent release parameters. This relates to a major issue with respect to co-transmission, namely how endogenous activation of modulatory systems regulates the differential release of co-localized transmitters. While exogenous application of substances or blocking uptake or breakdown mechanisms have provided useful information, it is a crude approach. We need to examine and mimic *in vivo* release to understand the functional effects of co-transmission. Optogenetics could greatly facilitate the analysis of these aspects by using specific activation patterns or optogenetic subcellular modifications to influence co-localization and co-release (see above).

We also need to understand the signals carried by the interactions of various co-transmitters. There interactions can generate additive, subtractive, non-linear or novel effects in different systems (Brezina, 2010; Harris-Warrick and Johnson, 2010; Nusbaum et al., 2017). There may be some logic to the interactions between particular transmitter systems, but we currently lack insight into this possibility. Exogenous application will not be without utility in addressing this aspect: even though we can optogentically activate neurons, teasing apart the interactive effects of co-transmitters will require precise control over what transmitters are released and in what concentrations, aspects facilitated by exogenous application. Even though exogenous application is a crude approach, fundamental insights have often relied on simplifying assumptions that generate testable hypotheses. But we must remember that insights obtained under simplifying conditions may not reflect how the system actually works.

In addition to normal release and interactions, we need to understand the influence of the functional state of targets, as state-dependent effects can influence neuromodulation. Understanding these changes will be important to understanding co-transmitter signaling. This will be especially important for treatments where the normal functional state is disturbed (e.g., spinal injury, Parkinson’s disease). This could also require understanding of how modulatory systems change after injury or in disease states (highlighted here by purinergic signaling and SCI). We cannot assume that a pathological state is the normal state with a missing component. The nervous system is adaptive, and widespread diaschisis-like or homeostatic changes clearly occur in response to perturbations.

Volume transmission is a recognized feature of neuronal signaling, especially for aminergic and peptidergic transmitters. In addition to the conditions that lead to the differential release or co-release of transmitters, volume signals can be modified as the transmitters move through the extracellular space by factors that can affect the breakdown of transmitters (potentially generating breakdown products that are biologically active). This seems to be conserved from invertebrates (e.g., Coleman et al., 1994; Wood et al., 2000; Duzzi et al., 2016) to mammals (e.g., Le Greves et al., 1985). Given that extracellular diffusion is likely to present a major factor affecting volume transmission, and given that the volume of the extracellular space can be modified by neuronal activity (Østby et al., 2009), there could be a circular interaction whereby transmitter effects change the neuronal activity that in turn affects transmitter effects. While not of obvious relevance, the extracellular space could thus be a major factor in understanding co- transmission.

A final point of obvious importance is that the effects of transmitter co-localization on behavior are still poorly understood. Classical approaches in the conventional model systems discussed here have examined cellular, synaptic and circuit level effects of transmitters and neuromodulation and related this to various aspects of behavior. However, the behavioral links often reflect assumptions and extrapolations rather than direct insight (e.g., fictive locomotion in spinal cord modulation). While links to actual locomotor behavior are often claimed, these can be tenuous, and where effects have been compared to those in intact systems they can differ (see above). The best hope of understanding behavioral effects seems to lie with optogenetic manipulations and molecular genetic approaches in mouse, *Drosophila*, and *C. elegans*. However, while we can analyze the effects of activating or manipulating modulatory systems on behavior in these systems, cellular and synaptic analyses are more difficult, making it more challenging to elucidate how the observed effects are mediated at the cellular or circuit levels. We need to develop ways to link modulation more directly to behavior in the classic model systems, and to examine physiological mechanisms in the more recently introduced genetically tractable systems. The conservation of effects between systems will help us to infer general cellular and behavioral principles across systems.

It is clear that co-transmission is a core signaling mechanism by which neurons in all nervous systems operate. Work in several model systems, including those reviewed here, has revealed some general principles, and supports a remarkable diversity of mechanisms resulting from co-transmission. However, challenges remain in establishing the roles of this basic design in the normal and dysfunctional operation of neurons, circuits and behavior, particularly in the mammalian CNS. We thus anticipate that the future will provide new, and often unexpected, insights in the roles of co-transmission in nervous system function.

## Author Contributions

ES wrote the “The *Aplysia* Feeding Circuit” and “Rodent Neocortex and Basal Ganglia” sections. JA-S wrote the “Hypothalamic Co-release” sections and commented on the manuscript. GB wrote the “Purinergic Co-transmission in the Autonomic and Central Nervous System” section, contributed to the “Historical Perspectives on Co-transmission” section, and commented on the manuscript. MN wrote the “Co-transmission Consequences in the Decapod Crustacean Stomatogastric Nervous System” section and commented on the manuscript. DP wrote the “Introduction” and “Conclusions”, the “Historical Perspectives on Co-transmission” section, and the “Spinal Cord Modulation and Co-transmission” section and commented on the manuscript. HS contributed to the “Rodent Neocortex and Basal Ganglia” section. Correspondence should be directed to the author of the relevant sections.

## Conflict of Interest Statement

The authors declare that the research was conducted in the absence of any commercial or financial relationships that could be construed as a potential conflict of interest.
